# System Pharmacology-Based Strategy to Decode the Synergistic Mechanism of Zhi-zhu Wan for Functional Dyspepsia

**DOI:** 10.3389/fphar.2018.00841

**Published:** 2018-08-06

**Authors:** Chun Wang, Qing Ren, Xue-Tong Chen, Zhi-Qian Song, Zhang-Chi Ning, Jia-He Gan, Xin-Ling Ma, Dong-Rui Liang, Dao-Gang Guan, Zhen-Li Liu, Ai-Ping Lu

**Affiliations:** ^1^Institute of Basic Theory for Chinese Medicine, China Academy of Chinese Medical Sciences, Beijing, China; ^2^Institute of Integrated Bioinformedicine and Translational Science, School of Chinese Medicine, Hong Kong Baptist University, Hong Kong, Hong Kong; ^3^Center of Bioinformatics, College of Life Science, Northwest A & F University, Yangling, China

**Keywords:** Zhi-zhu Wan, Zhishi, Baizhu, functional dyspepsia, therapeutic mechanism, system pharmacology

## Abstract

Functional dyspepsia (FD) is a widely prevalent gastrointestinal disorder throughout the world, whereas the efficacy of current treatment in the Western countries is limited. As the symptom is equivalent to the traditional Chinese medicine (TCM) term “stuffiness and fullness,” FD can be treated with Zhi-zhu Wan (ZZW) which is a kind of Chinese patent medicine. However, the “multi-component” and “multi-target” feature of Chinese patent medicine makes it challenge to elucidate the potential therapeutic mechanisms of ZZW on FD. Presently, a novel system pharmacology model including pharmacokinetic parameters, pharmacological data, and component contribution score (CS) is constructed to decipher the potential therapeutic mechanism of ZZW on FD. Finally, 61 components with favorable pharmacokinetic profiles and biological activities were obtained through ADME (absorption, distribution, metabolism, and excretion) screening *in silico*. The related targets of these components are identified by component targeting process followed by GO analysis and pathway enrichment analysis. And systematic analysis found that through acting on the target related to inflammation, gastrointestinal peristalsis, and mental disorder, ZZW plays a synergistic and complementary effect on FD at the pathway level. Furthermore, the component CS showed that 29 components contributed 90.18% of the total CS values of ZZW for the FD treatment, which suggested that the effective therapeutic effects of ZZW for FD are derived from all active components, not a few components. This study proposes the system pharmacology method and discovers the potent combination therapeutic mechanisms of ZZW for FD. This strategy will provide a reference method for other TCM mechanism research.

## Introduction

Functional dyspepsia (FD) is the pain or discomfort of the upper digestive tract without organic pathology that readily explains symptoms (Tack and Talley, 2013; Talley, 2016). The prevalence of FD in the general population is as high as 12–15% (El-Serag and Talley, 2004; Talley, 2016), and it significantly affects our moods and reduces the quality of life (Brun and Kuo, 2010). Treatments of FD involves eradication of *Helicobacter pylori* (Mokhtare et al., 2017), acid inhibition with proton pump inhibitors, tricyclic antidepressants (Ford et al., 2017), and prokinetic drugs (Quigley, 2017). Unfortunately, meta-analyses emphasized that these medications are still unsatisfactory for promoting the symptoms of FD, and the efficacy of currently available treatments be limited (Vakil et al., 2017). Clinical reports indicate that the safety and effectiveness of the Zhi-zhu Wan (ZZW) in the treatment of FD are remarkable.

ZZW is composed of two herbs, Zhishi (the immature fruit of *Citrus aurantium* L. or *Citrus sinensis* Osbeck) and Baizhu (the roots of *Atractylodes macrocephala* Koidz), which has prominence effect with FD (Wang et al., 2012; Xia et al., 2012), and their promotion of the gastrointestinal peristalsis activity has been confirmed in animal experiments (Liu, 2007; Huang et al., 2012; Chen J. et al., 2016). Baizhu showed the bidirectional regulation effects on gastrointestinal that might be related to the level of vasoactive intestinal peptide (VIP) and p substance (SP) (Chen J. et al., 2016). The combination of Zhishi and Baizhu may exert its therapeutic effects on FD by regulating the function of M and D endocrine cell, increasing the expression of acetylcholine and nitrogen monoxide, and regulating the gene expression of gut hormone receptor (Liu, 2007).

In pharmacokinetic studies, the pharmacokinetics and pharmacodynamics characteristics of ZZW after oral administration indicated that hesperidin and naringenin might be destroyed in the intestinal tract, metabolized by intestinal microflora, and excreted from bile or urine (Sun et al., 2013). In pharmacologic studies, flavonoids in Zhishi have a dose-dependent diastolic effect on pyloric circular smooth muscle strips in rats. These studies confirmed that the Zhishi and Baizhu could be beneficial in the treatment of patients with FD. Nevertheless, there is no literature expounds the underlying therapeutic mechanism of ZZW so far.

Considering the flaws of traditional experimental methods its approaches are difficult to reveal the co-module association mechanism of herb-component-gene-disease due to the “multi-component” and “multi-target” features of the TCM systems. Systemic pharmacology is an effective tool to elucidate the synergistic and potential mechanisms of the networks between component-target and target-disease, it provides a new perspective on the therapeutic mechanisms of TCM. Recently, several system pharmacology models were used to decode the underlying mechanism of herb pair (Cheng S. P. et al., 2016; Zhang et al., 2016; Yue et al., 2017) and Chinese formulae (Zhang et al., 2015), but most of them losts the synergistic information.

**Figure 1:**
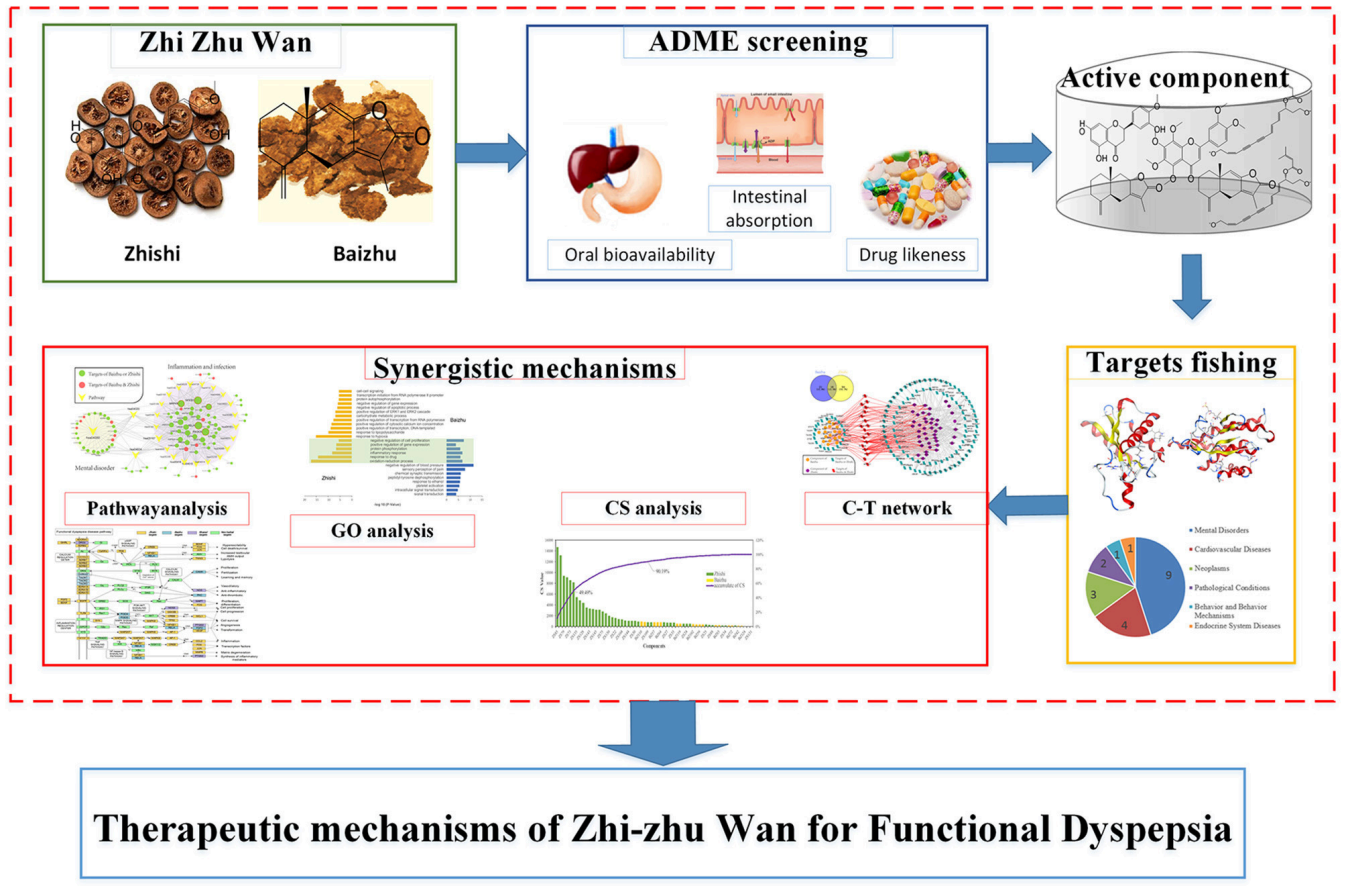
The work scheme of system pharmacology approach.

Currently, a novel system pharmacology model is developed to explore the therapeutic mechanism of ZZW in the treatment of FD (Figure 1), integrating pharmacokinetics synthesis screening, target identification and network analysis. Specifically, four parameters are used for ADME (absorption, distribution, metabolism, and excretion) screening to ensure more comprehensive first. Subsequently, the target from docking database and reference database are both retrieved to ensure the accuracy and effectiveness of the component-target (C-T) network. Ultimately, the network analysis combined with contribution score (CS) are used to elucidate the synergistic molecular actions of Zhishi-Baizhu. Hopefully, these results will provide a strategy for illuminating the therapeutic mechanism of TCM at molecular level.

## Methods

### Chemical components database

All components of ZZW were collected from five publicly available natural product data sources: TCMSP database (http://lsp.nwu.edu.cn/index.php), Shanghai Institute of Organic Chemistry, Chinese Academy of Sciences. Chemistry Database [DB/OL] (http://www.organchem.csdb.cn [1978-2018], Traditional Chinese Medicine integrated database (TCMID, http://www.megabionet.org/tcmid/), Traditional Chinese Medicine database@Taiwan (TCM@Taiwan, http://tcm.cmu.edu.tw/zh-tw), and TCM-MESH (http://mesh.tcm.microbioinformatics.org/). For all components, using Open Babel toolkit (version 2.4.1) to convert the initial structure formats (e.g., mol2) to the unified SDF format. Subsequently, the properties of components were retrieved from TCMSP, including molecular weight (MW), oral bioavailability (OB), Caco-2 permeability (Caco-2), drug-likeness (DL), Moriguchi octanol-water partition coefficient (LogP) (MLogP), number of acceptor atoms for H-bonds (nHAcc), number of donor atoms for H-bonds (nHDon), and topological polar surface area (TPSA), and GI absorption was retrieved from SwissADME (http://www.swissadme.ch/index.php).

### ADME screening

In modern drug discovery, early assessment of absorption, distribution, metabolism, and excretion (ADME) screening has become an essential process. The proper use of ADME results can give preference to those drug candidates that are more likely to have good pharmacokinetic properties and minimize potential drug-drug interactions (Wang J. H. et al., 2017). In the present work, four ADME-related models, including OB, Caco-2, DL, and GI absorption were employed to screen the active components from ZZW (Figure S1).

OB (%F) depicts the percentage of an orally administered dose of the chemical components in herbs that reaches the systemic circulation, which displays the convergence of the ADME process. A robust *in silico* system OBioavail 1.1 (Xu et al., 2012) was performed to calculate the OB values of all components in ZZW. Those components with suitable OB ≥ 30% were selected as candidate components for further research.

Human intestinal cell line Caco-2 is generally employed to study the passive diffusion of drugs across the intestinal epithelium, the transport rates of components (nm/s) in Caco-2 monolayers represents the intestinal epithelial permeability in TCMSP (Ru et al., 2014). The Caco-2 value of the components in ZZW was obtained from TCMSP (http://lsp.nwu.edu.cn/tcmsp.php). Compounds with Caco-2 > −0.4 were selected as candidate components, because components with Caco-2 < −0.4 are not permeable.

DL is an established concept for drug design that is used to estimate which compounds have the “drug-like” prospective. The DL values of these components were calculated by the database-dependent DL evaluation approach based on Tanimoto coefficient, which is expressed as *T* (*A, B*) = (*A* × *B*)/(|*A*|^2^ + |*B*|^2^ − *A* × *B*). In this equation, A represents the molecular descriptor of herbal components, and B is the average molecular property of all components in Drugbank. The threshold of DL was set to 0.18, which is used as a selection criterion for “drug-like” compounds in the traditional Chinese herbs (Tao et al., 2013). During the screening process of Baizhu, we found that the DL value of lactones was lower than 0.18 but higher than 0.14, Considering lactones are the main active and characteristic compounds in BZ (China, 2015), so the screening criterion of Baizhu was defined as DL ≥ 0.14.

GI absorption is a pharmacokinetic behavior crucial to estimate at various stages of the drug discovery processes, which can be calculated by an accurate predictive model, IntestinaL EstimateD permeation method (BOILED-Egg) (Daina and Zoete, 2016). The GI absorption value of the components in ZZW was obtained from SwissADME (http://www.swissadme.ch/index.php) (Daina et al., 2017). The screening criterion of GI absorption was defined as high.

### Targets identification

To obtain the target of active components in ZZW, the commonly used databases, i.e., HitPick (Liu et al., 2013), Similarity Ensemble Approach (SEA) (Keiser et al., 2007), STITCH (Szklarczyk et al., 2016), and Swiss Target Prediction (Gfeller et al., 2014), were employed to identify the targets. All chemical structures were prepared and converted into canonical SMILES using Open Babel Toolkit (version 2.4.1). In addition, the target results were confirmed by literature reviews. Sequently, to anatomize the role of ZZW in the treatment of FD, the relationship between the obtained targets and diseases was calculated using the hypergeometric distribution algorithm:

$$P_{\left(\text{ZZW},\;d\right)}=1-\sum_{i=0}^{k-1}\frac{\left(\frac{K}{i}\right)\left(\frac{N-K}{n-i}\right)}{\left(\frac{N}{n}\right)}$$

where *N* is the total number of targets in DisGeNET (Piñero et al., 2017), *K* is the number of targets associated with disease *d*, *n* is the quantity about the targets of ZZW, k is the number of targets shared by ZZW and disease *d*. *P*-value indicates the consequence of relevance between ZZW and disease *d* (significant when *P* < 0.05).

### Gene ontology and pathway analysis

To analyze the main function of the target genes, Gene Ontology (GO) analysis was performed using the Diversity Visualization Integrated Database (DAVID 6.8) (Huang et al., 2009). The false discovery rate (FDR) (Dupuy et al., 2007) was calculated to correct the *p*-value. The criterion for difference screening was FDR < 0.05.

The latest pathway data were obtained from the Kyoto Encyclopedia of Genes and Genomes (KEGG) database (Draghici et al., 2007) for KEGG pathway enrichment analyses. *P*-values were set at 0.05 as the cut-off criterion. The results of analysis were annotated by Pathview (Luo and Brouwer, 2013) in the R Bioconductor package (https://www.bioconductor.org/).

### Networks construction

The component-target network was established to find the key target. Then, the target-pathway (T-P) network was constructed to find out the relationship between the target and pathway. Cytoscape 3.5.1 (Shannon et al., 2003), an open-source software platform for visualizing complex networks, was employed to visualize the networks.

### Contribution score calculation

To estimate the effect of each component of ZZW on FD treatment, we established a mathematical formula:

(1) $$A_{ij}=\;\omega_{ei}+\left|\frac{C_{Ai}+C_{Bi}}{C_{Ai}-C_{Bi}}\right|$$

(2) $$\omega_{ei}=\frac{C_{edge}}{T_{edge}}$$

(3) $$CS\left(i\right)=\sum_{ij}^{n}C_{i}\times \left[A_{ij}\times P_{j}\right]$$

Where *i* is the number of components and *j* is the number of proteins.

The contribution score *(CS)* represents the network contribution of one component and its effectiveness in FD. *C* represents the degree of each component, *P* represents the degree of each protein, which is calculated by Cytoscape 3.5.1. *C*_*Ai*_ represents the degree of each component only in Zhishi *C-T* network, and *C*_*Bi*_ represents the degree of each component only in Baizhu *C-T* network. *A*_*ij*_ is the index of affinity determined from the ω_*ei*_ value.

### Side effect prediction

Side effect information was obtained from SIDER, which accumulates reported side effects from package inserts for marketed drugs (Kuhn et al., 2010), To encode drug chemical structures, a fingerprint was used, which consisted of 61 chemical substructures defined in the PubChem database (Li et al., 2010). This resulted in a binary profile referred to as chemical substructure profile. The side effect prediction use the Ordinary canonical correlation analysis (OCCA) framework (Mizutani et al., 2012).

### Statistical analysis

To compare the molecular properties of all components in Zhishi and Baizhu, SPSS22.0 was used for statistical analysis. Data were analyzed using the student's *t-*test for comparison. When *P* < 0.05, the differences were considered statistically significant.

## Results

Based on a system pharmacology model, the therapeutic mechanisms of FD by ZZW were elucidated. All ZZW compounds were collected from database and literature. Next, the ADME method was used to screen for potential active components. Then related targets, disease, and pathway were identified from integrated predictive models. The obtained data were used to construct C-T and T-P networks, respectively. Finally, the CS of all compounds was calculated to illustrate the combination mechanism.

### Components comparisons in zhishi and baizhu

By a systematic search of the public databases, a total of 378 components were retrieved in Zhishi (150) and Baizhu (128). Interestingly, the species of components in Zhishi and Baizhu are different, the major components of Zhishi are flavonoids and volatile oil, whereas Baizhu is lactones and volatile oil. The detail information of these components was provided in Table S1.

**Figure 2:**
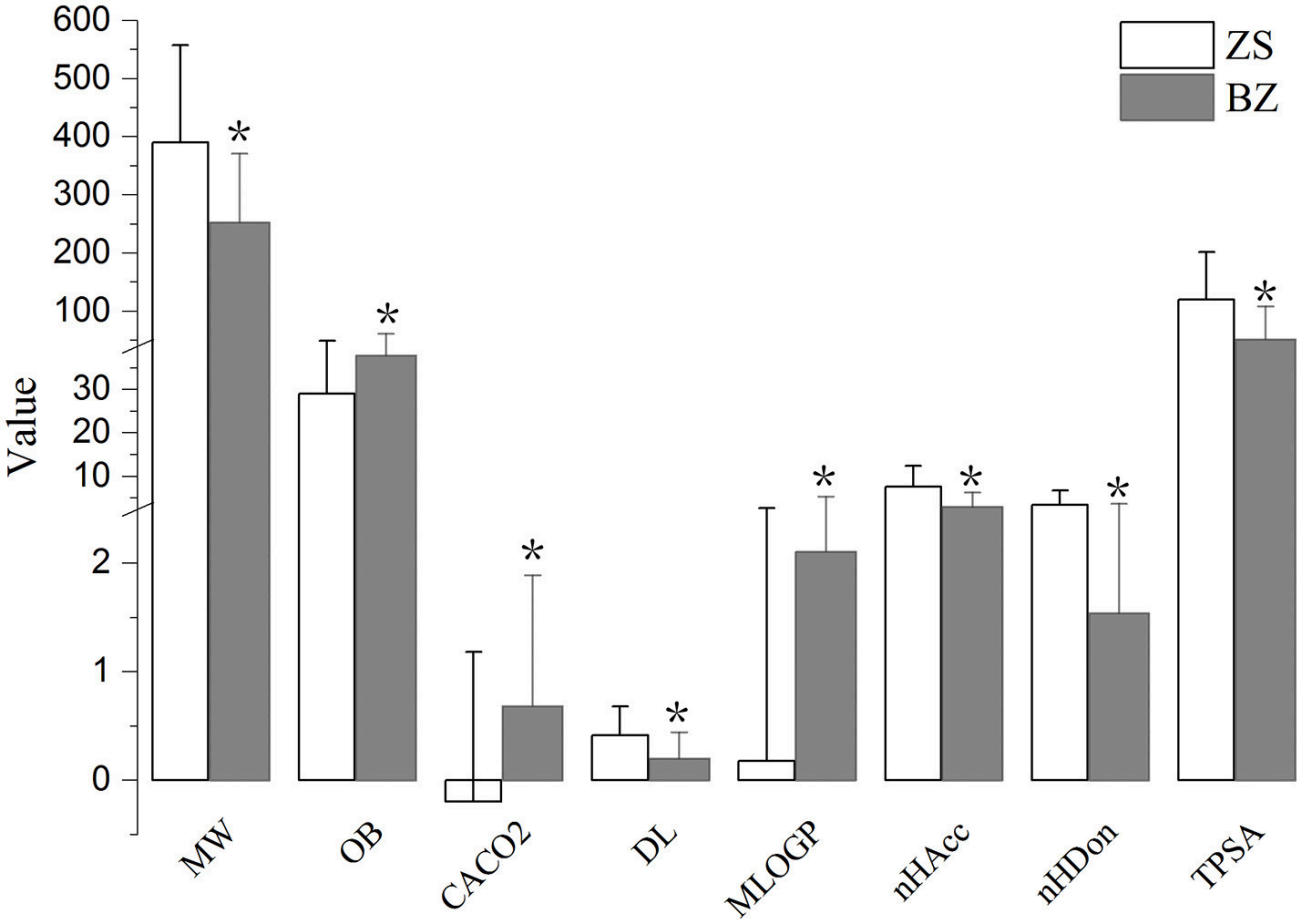
The molecular properties of all components in Zhishi and Baizhu. Molecular properties including molecular weight (MW), oral bioavailability (OB), Caco-2 permeability (Caco-2), drug-likeness (DL), Moriguchi octanol-water partition coeff. Log P (MlogP), number of acceptor atoms for H-bonds (nHAcc), number of donor atoms for H-bonds (nHDon), and topological polar surface area (TPSA). *P < 0.01 by two tailed *t-*test (vs. Zhishi).

To further describe the differences from the components of Zhishi and Baizhu, nine properties of these components were compared, including MW, MLogP, nHDon, nHAcc, OB, Caco-2 permeability, DL, TPSA, and GI absorption. As shown in Figure 2, the eight value of the components in Zhishi and Baizhu were significantly different (*P* < 0.01) but the majority of the components did not violate Lipinski's rule of five (Lipinski et al., 2001). (1) For MW, the average value of components in Zhishi (393.39) is significantly higher than that in Baizhu (252.67) (*P* = 7.20E-15). (2) For bioavailability, the average OB value of Zhishi (28.94) is lower than that of Baizhu (37.76) (*P* = 9.72E-4). (3) For permeability, the average Caco-2 value of Zhishi (−0.20) is significally lower than that of Baizhu (0.69) (*P* = 2.26E-08). (4) For DL, unlike OB and Caco-2, Zhishi possessed higher average DL value (0.41), that is very different from that of Baizhu (0.20) (*P* = 1.58E-11). (5) Compared with the components of Zhishi (0.15), the MLogP value of Baizhu exhibited siginifically higher average MLogP value (2.10) (*P* = 7.58E-08), which indicates the majority components in Baizhu are hydrotropic, but that in Zhishi are hydrophobic. (6) The values of nHAcc, nHDon, and TPSA in Zhishi (7.67, 3.50, 51.65, respectively) are all higher than those in Baizhu (2.95, 1.54, 120.45, respectively) (6.71E-19, 1.81E-08, 1.54E-14, respectively).

All the results showed that there are differences between the components of Zhishi and Baizhu, which may be due to the distinct chemo-physical properties of the components from two herbs. Our results also showed that the components from Baizhu have better pharmacokinetic properties (OB and Caco-2), whereas the components in Zhishi have better DL. Although there are obvious difference of main components between Zhishi and Baizhu, the two herbs have the identical spleen-fortifying and digestion-promoting, qi-promoting and damp-dispelling effects, which may also elucidate why Zhishi-Baizhu can produce synergistic effects.

### Active components in zhishi-baizhu

Even though any TCM formulation contains multiple components, only a few components possess satisfactory pharmacodynamic and pharmacokinetic properties. In the current work, four ADME-related models, including OB, Caco-2, DL, and GI, were employed to screen for active components. After ADME screening, a few components that did not meet the four screening criteria were also selected because of their high amount and high bioactive. Therefore, 61 active components were filtered out of the 378 components of ZZW. The detail information was shown in Table 1. Additionally, we used Small Molecule Subgraph Detector (SMSD) Toolkit (Rahman et al., 2009) to calculate the drug similarity based on Tanimoto Coefficient, which was often used to predict Drug-drug Interations (DDIs) (Takeda et al., 2017), and found that in 1,891 pairs of similarity comparisons, the similarity of 1,018 pair <=0.2, account for 54% (Figure S3). In order to calculate the potential side effect of all active compounds, we employ the OCCA framework to predict the side effects and found the slight side effects were mainly focused on agitation, weakness, and dizziness (Figure S4 and Table S4).

**Table 1:** The information of active components in ZZW.

**ID**	**Molecule_name**	**MW**	**OB**	**Caco-2**	**DL**	**MLOGP**	**nHAcc**	**nHDon**	**TPSA**	**GI absorption**
BZ27	Atractylenolactam	229.32	56.48	1.23	0.15	2.85	1	1	29.1	High
BZ42	Anhydroatractylolide	234.34	52.24	1.24	0.15	3.44	2	0	26.3	High
BZ57	8β-methoxy-atractylenolide I	260.33	54.47	1.02	0.19	2.63	3	0	35.53	High
BZ59	14α-methyl butyryl-14-acetyl-2E,8E,10E-atractylentriol	316.40	64.50	0.20	0.23	2.71	4	2	66.76	High
BZ60	12α-methylbutyryl-14-acetyl-2E,8Z,10E-atractylentriol	358.43	62.69	0.41	0.29	3.07	5	1	72.83	High
BZ64	8β-ethoxyatractylenolide- II	276.38	56.48	1.08	0.21	3.37	3	0	35.53	High
BZ72	Isoasterolide A	232.32	52.65	1.27	0.15	3.35	2	0	26.3	High
BZ75	Atractylenolide VII	262.39	40.99	1.32	0.14	3.84	2	0	26.3	High
BZ83	Atractylodes macrocephala	462.68	45.96	0.85	0.81	2.47	3	1	46.53	High
BZ84	Biatractylolide	462.63	45.96	0.84	0.81	5.43	4	0	52.6	High
BZ100	8β-ethoxy atractylenolide-II	276.38	56.48	1.08	0.21	3.37	3	0	35.53	High
BZ102	Atractylenolide I	230.31	35.21	1.32	0.15	3.26	2	0	26.3	High
BZ107	Atractylone	216.324	25.99	1.74	0.13	3.42	1	0	13.14	High
BZ110	AtractylenolideII	232.32	43.54	1.31	0.15	3.35	2	0	26.3	High
BZ119	3β-acetoxyatractylone	274.36	34.74	1.19	0.22	2.83	3	0	39.44	High
BZ124	14-acetyl-12-senecioyl-2E,8Z,10E-atractylentriol	356.42	63.37	0.26	0.30	2.99	5	1	72.83	High
BZ125	Atractylenolide III	248.32	67.29	0.76	0.17	2.47	3	1	46.53	High
ZS21	8-geranyloxypsoralen	338.4	41.92	1.178	0.418	3.23	4	0	52.58	High
ZS22	5-Geranyloxy-7-Methoxycoumarin	328.4	44.23	1.121	0.300	3.16	4	0	48.67	High
ZS23	Bergamottin	338.4	41.73	1.161	0.421	3.23	4	0	52.58	High
ZS24	Phellopterin	300.31	37.43	0.978	0.279	1.82	5	0	61.81	High
ZS25	Isoimperatorin	270.28	47.54	1.057	0.225	2.14	4	0	52.58	High
ZS26	6′-7′-dihydroxybergamottin	372.41	70.77	0.12	0.52	1.66	6	2	93.04	High
ZS27	Epoxybergamottin	354.4	57.25	0.922	0.523	2.48	5	0	65.11	High
ZS28	Cnidilin	300.31	42.42	0.948	0.280	1.82	5	0	61.81	High
ZS30	Epoxyaurapten	314.38	62.78	0.952	0.309	2.74	4	0	51.97	High
ZS34	Byakangelicin	334.32	34.89	−0.01	0.35	0.29	7	2	102.27	High
ZS35	Heraclenol	304.29	72.63	0.08	0.29	0.57	6	2	93.04	High
ZS36	Oxypeucedanin hydrate	304.29	33.07	−0.06	0.29	0.57	6	2	93.04	High
ZS39	Isoponcimarin	330.37	63.28	0.534	0.313	1.91	5	0	69.04	High
ZS40	Poncimarin	330.37	79.20	0.754	0.350	1.99	5	0	64.5	High
ZS41	Byakangelicol	316.31	45.21	0.760	0.356	1.08	6	0	74.34	High
ZS42	Oxypeucedanin	286.28	66.18	0.870	0.297	1.39	5	0	65.11	High
ZS71	Monohydryoxy-tetramethoxyflavone	358.34	45.38	1.19	0.37	0.4	7	1	87.36	High
ZS73	Diosmetin	300.26	42.87	0.46	0.27	0.22	6	3	100.13	High
ZS75	5-demethylnobiletin	388.37	89.03	1.01	0.48	0.11	8	1	96.59	High
ZS79	Chrysoeriol	300.26	41.60	0.45	0.27	0.22	6	3	100.13	High
ZS82	Sakuranetin	286.28	40.19	0.59	0.24	0.96	5	2	75.99	High
ZS85	Acacetin	284.26	37.69	0.65	0.24	0.77	5	2	79.9	High
ZS86	Isosakuranetin	286.28	37.59	0.58	0.24	0.96	5	2	75.99	High
ZS88	N-methyl tyramine-O-alpha-L-rhamnopyranoside	297.35	36.70	−0.04	0.19	−0.16	6	4	91.18	High
ZS104	Synephrine	167.21	75.25	0.63	0.04	0.65	3	3	52.49	High
ZS105	4-[(2S,3R)-5-[(E)-3-hydroxyprop-1-enyl]-7-methoxy-3-methylol-2,3-dihydrobenzofuran-2-yl]-2-methoxy-phenol	358.39	50.76	0.03	0.39	1.09	6	3	88.38	High
ZS107	5,7,4′-Trimethylapigenin	312.32	39.83	1.01	0.3	1.25	5	0	57.9	High
ZS108	Hesperetin	302.28	47.74	0.28	0.27	0.41	6	3	96.22	High
ZS109	6-Methoxy aurapten	328.4	31.24	1.01	0.3	3.16	4	0	48.67	High
ZS110	Ammidin	270.28	34.55	1.13	0.22	2.14	4	0	52.58	High
ZS115	Naringenin	272.26	59.29	0.28	0.21	0.71	5	3	86.99	High
ZS117	Tetramethoxyluteolin	342.34	43.68	0.96	0.37	0.94	6	0	67.13	High
ZS123	Prangenin	286.28	43.60	0.8	0.29	1.39	5	0	65.11	High
ZS128	Eriodyctiol (flavanone)	288.25	41.35	0.05	0.24	0.16	6	4	107.22	High
ZS130	Hesperidin	610.56	13.33	−2.03	0.67	−3.04	15	8	234.29	Low
ZS131	Isolimonic acid	639.01	48.86	0.43	0.18	4.33	3	1	57.61	High
ZS134	Isosinensetin	372.37	51.15	1.16	0.44	0.63	7	0	76.36	High
ZS135	Sinensetin	372.37	50.56	1.12	0.45	0.63	7	0	76.36	High
ZS137	Luteolin	286.24	36.16	0.19	0.25	−0.03	6	4	111.13	High
ZS143	Naringin	580.53	6.92	−1.99	0.78	−2.77	14	8	225.06	Low
ZS144	Narirutin	580.53	8.15	−1.8	0.75	−2.77	14	8	225.06	Low
ZS145	Neohesperidin_qt	302.28	71.17	0.26	0.27	0.41	6	3	96.22	High
ZS146	Nobiletin	402.39	61.67	1.05	0.52	0.34	8	0	85.59	High
ZS149	Prangenin hydrate	304.29	72.63	0.14	0.29	0.57	6	2	93.04	High
ZS150	Neohesperidin	610.62	11.57	−2.05	0.69	−3.04	15	8	234.29	Low

### Active components from zhishi

Through ADME screening, 44 out of 150 components were selected from Zhishi, and most of them have ideal pharmacokinetic profiles. For example, hesperetin (ZS108, OB = 47.74%, Caco-2 = 0.28, DL = 0.27, GI = high) exhibits antioxidants (de Souza et al., 2016), anti-inflammatory(Choi and Lee, 2010), and vasoprotective (Kumar et al., 2013) actions; Similarly, naringenin (ZS115, OB = 59.29%, Caco-2 = 0.28, DL = 0.21, GI = high) has anti-inflammatory (Manchope et al., 2017), antibacterial (Wang L. H. et al., 2017), neuroprotective(Ramakrishnan et al., 2016) effects. It is worth noting that the value of Caco-2 in dihydroflavonosides of Zhishi is lower, such as narirutin (ZS144), naringin (ZS143), hesperidin (ZS130), and neohesperidin (ZS150), however, the four flavonoids were the main bioactive components in Zhishi and exhibited relatively high abundances (Liu et al., 2012), so these components were also preserved. Especially, the value of DL in synephrine (ZS104) is low, but it is the marker components for quality control of Zhishi in Chinese Pharmacopeia (China, 2015). For the above reasons, 44 components were considered as potential active components of Zhishi (Table 1).

### Active components from baizhu

Among 131 components in BZ, 17 components meet the screening criteria. For instance, atractylenolide I, II, III (BZ102, OB = 35.21%, Caco-2 = 1.32, DL = 0.15, GI = high; BZ110, OB = 43.54%, Caco-2 = 1.31, DL = 0.15, GI = high; BZ125, OB = 67.29%, Caco-2 = 0.76, DL = 0.17, GI = high) was the quality marker of BZ in *Chinese Pharmacopeia* (China, 2015) and has anti-inflammatory (Ji et al., 2016), anticoagulation effect (Tang et al., 2017) gastrointestinal repair effects (Song et al., 2017); Atractylenolactam (BZ27, OB = 56.48%, Caco-2 = 1.23, DL = 0.15, GI = high) exhibits anti-inflammatory activity (Hoang et al., 2016); Biatractylolide (BZ84, OB = 45.96%, Caco-2 = 0.84, DL = 0.81, GI = high) has a neuroprotective effect on glutamate-induced injury in PC12 and SH-SY5Y cells (Zhu et al., 2017). Specially, atractylone has been showed to have anti-microbial and anti-inflammatory activities (Sin et al., 1989), so it was also regarded to be active components. The detail information of 17 components was showed in Table 1.

### Target proteins of zhishi-baizhu

To determine the relationship between the target and FD, we collected disease targets and used a hypergeometric distribution to describe the relationship probability between targets and diseases. It's worth noting that the target of active components is related to FD (*p* < 0.05). In addition, the active components-related targets were further compared with all other disease in DisGeNET and the final relationship was ranked by the P value. Among the top 20 diseases, 9 were mental disorder (Figure S2 and Table S3) which is one of the pathogenic factors of FD that confirmed by recent studies (Aro et al., 2015). Overall, most targets are related with FD, which indicated that ZZW can be used to treat FD.

**Figure 3:**
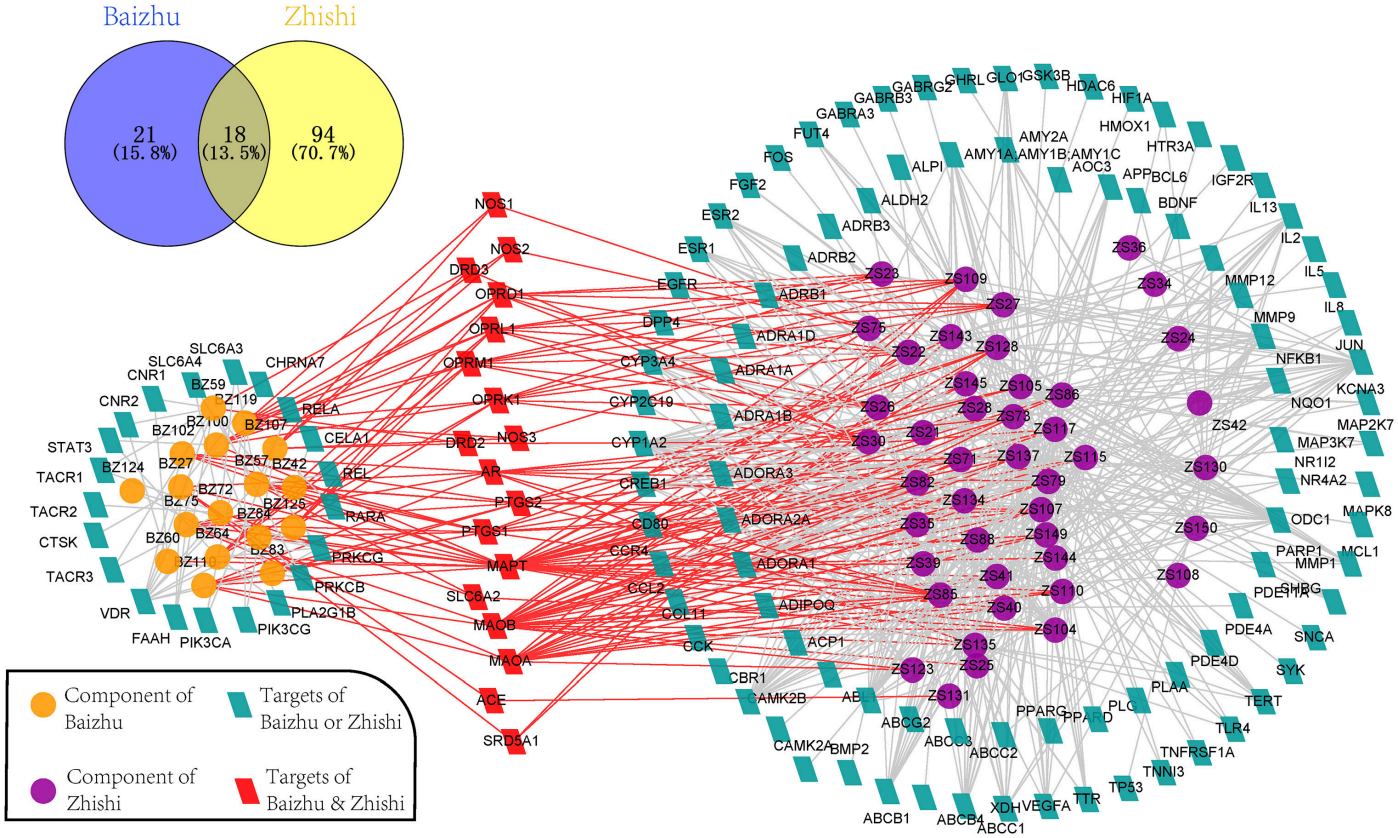
Component-target network of ZZW. The orange and purple ellipse nodes are active components of Baizhu and Zhishi, and the blue parallelogram nodes are the related targets, while the red parallelogram nodes are the same targets of Zhishi and Baizhu.

To explore the therapeutic mechanism of ZZW in the treatment of FD, 61 active components and 133 targets (Table 2) were used to construct the C-T network (Figure 3). Several of these active components are related multiple targets, resulting in 650 component-target associations between 61 active components and 133 targets. The average number of targets per component is 10.6, and the mean degree of components per target is 4.9, it shows that ZZW handles multi-component and multi-target characteristics of ZZW for treating FD. Acacetin (ZS85, degree = 38) has the highest number of targets, followed by luteolin (ZS137, degree = 36), chrysoeriol (ZS79, degree = 30), and 5,7,4′-Trimethylapigenin (ZS107, degree = 28), demonstrating the crucial roles of these components in the treatment of FD.

**Table 2:** The information of the related targets of ZZW.

**Gene**	**Protein name**	**Uniprot ID**
ABCB1	Multidrug resistance protein 1	P08183
ABCB4	Multidrug resistance protein 3	P21439
ABCC1	Multidrug resistance-associated protein 1	P33527
ABCC2	Canalicular multispecific organic anion transporter 1	Q92887
ABCC3	Canalicular multispecific organic anion transporter 2	O15438
ABCG2	ATP-binding cassette sub-family G member 2	Q9UNQ0
ABL1	Tyrosine-protein kinase ABL1	P00519
ACE	Angiotensin-converting enzyme	P12821
ACP1	Low molecular weight phosphotyrosine protein phosphatase	P24666
ADIPOQ	Adiponectin	Q15848
ADORA1	Adenosine receptor A1	P30542
ADORA2A	Adenosine receptor A2a	P29274
ADORA3	Adenosine receptor A3	P33765
ADRA1A	Alpha-1A adrenergic receptor	P35348
ADRA1B	Alpha-1B adrenergic receptor	P35368
ADRA1D	Alpha-1D adrenergic receptor	P25100
ADRB1	Beta-1 adrenergic receptor	P08588
ADRB2	Beta-2 adrenergic receptor	P07550
ADRB3	Beta-3 adrenergic receptor	P13945
ALDH2	Aldehyde dehydrogenase, mitochondrial	P05091
ALPI	Intestinal-type alkaline phosphatase	P09923
AMY1A	Alpha-amylase 1	P04745
AMY2A	Pancreatic alpha-amylase	P04746
AOC3	Membrane primary amine oxidase	Q16853
APP	Amyloid-beta A4 protein	P05067
AR	Androgen receptor	P10275
BCL6	B-cell lymphoma 6 protein	P41182
BDNF	Brain-derived neurotrophic factor	P23560
BMP2	Bone morphogenetic protein 2	P12643
CAMK2A	Calcium/calmodulin-dependent protein kinase type II subunit alpha	Q9UQM7
CAMK2B	Calcium/calmodulin-dependent protein kinase type II subunit beta	Q13554
CBR1	Carbonyl reductase [NADPH] 1	P16152
CCK	Cholecystokinin	P06307
CCL11	Eotaxin	P51671
CCL2	C-C motif chemokine 2	P13500
CCR4	C-C chemokine receptor type 4	P51679
CD80	T-lymphocyte activation antigen CD80	P33681
CELA1	Chymotrypsin-like elastase family member 1	Q9UNI1
CHRNA7	Neuronal acetylcholine receptor subunit alpha-7	P36544
CNR1	Cannabinoid receptor 1	P21554
CNR2	Cannabinoid receptor 2	P34972
CREB1	Cyclic AMP-responsive element-binding protein 1	P16220
CTSK	Cathepsin K	P43235
CYP1A2	Cytochrome P450 1A2	P05177
CYP2C19	Cytochrome P450 2C19	P33261
CYP3A4	Cytochrome P450 3A4	P08684
DPP4	Dipeptidyl peptidase 4	P27487
DRD2	D(2) dopamine receptor	P14416
DRD3	D(3) dopamine receptor	P35462
EGFR	Epidermal growth factor receptor	P00533
ESR1	Estrogen receptor	P03372
ESR2	Estrogen receptor beta	Q92731
FAAH	Fatty-acid amide hydrolase 1	O00519
FGF2	Fibroblast growth factor 2	P09038
FOS	Proto-oncogene c-Fos	P01100
FUT4	Alpha-(1,3)-fucosyltransferase 4	P22083
GABRA3	Gamma-aminobutyric acid receptor subunit alpha-3	P34903
GABRB3	Gamma-aminobutyric acid receptor subunit beta-3	P28472
GABRG2	Gamma-aminobutyric acid receptor subunit gamma-2	P18507
GHRL	Appetite-regulating hormone	Q9UBU3
GLO1	Lactoylglutathione lyase	Q9UBU3
GSK3B	Glycogen synthase kinase-3 beta	P49841
HDAC6	Histone deacetylase 6	Q9UBN7
HIF1A	Hypoxia-inducible factor 1-alpha	Q16665
HMOX1	Heme oxygenase 1	P09601
HTR3A	5-hydroxytryptamine receptor 3A	P46098
IGF2R	Cation-independent mannose-6-phosphate receptor	P11717
IL13	Interleukin-13	P35225
IL2	Interleukin-2	P60568
IL5	Interleukin-5	P05113
IL8	Interleukin-8	P10145
JUN	Transcription factor AP-1	P05412
KCNA3	Potassium voltage-gated channel subfamily A member 3	P22001
MAOA	Amine oxidase [flavin-containing] A	P21397
MAOB	Amine oxidase [flavin-containing] B	P27338
MAP2K7	Dual specificity mitogen-activated protein kinase kinase 7	O14733
MAP3K7	Mitogen-activated protein kinase kinase kinase 7	O43318
MAPK8	Mitogen-activated protein kinase 8	P45983
MAPT	Microtubule-associated protein tau	P10636
MCL1	Induced myeloid leukemia cell differentiation protein Mcl-1	Q07820
MMP1	22 kDa interstitial collagenase	P03956
MMP12	Macrophage metalloelastase	P39900
MMP9	67 kDa matrix metalloproteinase-9	P14780
NFKB1	Nuclear factor NF-kappa-B p105 subunit	P19838
NOS1	Nitric oxide synthase, brain	P29475
NOS2	Nitric oxide synthase, inducible	P35228
NOS3	Nitric oxide synthase, endothelial	P29474
NQO1	NAD(P)H dehydrogenase [quinone] 1	P15559
NR1I2	Nuclear receptor subfamily 1 group I member 2	O75469
NR4A2	Nuclear receptor subfamily 4 group A member 2	P43354
ODC1	Ornithine decarboxylase	P11926
OPRD1	Delta-type opioid receptor	P41143
OPRK1	Kappa-type opioid receptor	P41145
OPRL1	Nociceptin receptor	P41146
OPRM1	Mu-type opioid receptor	P35372
PARP1	Poly [ADP-ribose] polymerase 1	P09874
PDE11A	Dual 3′,5′-cyclic-AMP and -GMP phosphodiesterase 11A	Q9HCR9
PDE4A	cAMP-specific 3′,5′-cyclic phosphodiesterase 4A	P27815
PDE4D	cAMP-specific 3′,5′-cyclic phosphodiesterase 4D	Q08499
PIK3CA	Phosphatidylinositol 4,5-bisphosphate 3-kinase catalytic subunit alpha isoform	P42336
PIK3CG	Phosphatidylinositol 4,5-bisphosphate 3-kinase catalytic subunit gamma isoform	P48736
PLA2G1B	Phospholipase A2	P04054
PLAA	Phospholipase A-2-activating protein	Q9Y263
PLG	Plasmin light chain B	P00747
PPARD	Peroxisome proliferator-activated receptor delta	Q03181
PPARG	Peroxisome proliferator-activated receptor gamma	P37231
PRKCB	Protein kinase C beta type	P05771
PRKCG	Protein kinase C gamma type	P05129
PTGS1	Prostaglandin G/H synthase 1	P23219
PTGS2	Prostaglandin G/H synthase 2	P35354
RARA	Retinoic acid receptor alpha	P10276
REL	Proto-oncogene c-Rel	Q04864
RELA	Transcription factor p65	Q04206
SHBG	Sex hormone-binding globulin	P04278
SLC6A2	Sodium-dependent noradrenaline transporter	P23975
SLC6A3	Sodium-dependent dopamine transporter	Q01959
SLC6A4	Sodium-dependent serotonin transporter	P31645
SNCA	Alpha-synuclein	P37840
SRD5A1	3-oxo-5-alpha-steroid 4-dehydrogenase 1	P18405
STAT3	Signal transducer and activator of transcription 3	P40763
SYK	Tyrosine-protein kinase SYK	P43405
TACR1	Substance-P receptor	P25103
TACR2	Substance-K receptor	P21452
TACR3	Neuromedin-K receptor	P29371
TERT	Telomerase reverse transcriptase	O14746
TLR4	Toll-like receptor 4	O00206
TNFRSF1A	Tumor necrosis factor receptor superfamily member 1A	P19438
TNNI3	Troponin I, cardiac muscle	P19429
TP53	Cellular tumor antigen p53	P04637
TTR	Transthyretin	P02766
VDR	Vitamin D3 receptor	P11473
VEGFA	Vascular endothelial growth factor A	P15692
XDH	Xanthine dehydrogenase/oxidase	P47989

In Zhishi, 112 target proteins are identified for 44 active components with 538 interactions. The causes of FD mainly include dyspepsia, *Helicobacter pylori* infection, depression, etc. (Talley, 2016), which can generate inflammation, gastrointestinal movement dysfunction, and etc. Intriguingly, most of targets of the components in Zhishi are related to inflammation and gastrointestinal peristalsis. For instance, the three components of Zhishi, including ZS39, ZS108, and ZS143, may interact with PPARA and PPARG, which are members of a subfamily of the nuclear receptors and can modulate inflammatory responses (Varga et al., 2011). The other six active components, ZS71, ZS85, ZS105, ZS107, ZS128, and ZS134, were identified as interacting with PTGS1and PTGS2, also known as COX-1 and COX-2, COX-1 is a constitute engine expressed in most tissues including blood platelets and at any site of inflammation and promotes the production of natural mucus lining that protects the inner stomach, whereas COX-2 is involved in pain produced by inflammation (Mandlik et al., 2015). Furthermore, we have found that five components (ZS73, ZS79, ZS115, ZS117, and ZS145) are related to ABCB1 and ABCC1-3, which may critically participate in the protection of the intestinal barrier by excluding drugs, nutrients, or bacterial compounds back into the gut lumen (Langmann et al., 2004).

In Baizhu, 39 target proteins are identified for 17 active components with 112 interactions, including MAOA, MAOB, NOS1-3, TACR1, SLC6A4, STAT3, etc. Interestingly, majority of them are related to mental disorders and inflammation, which are confirmed associated with the pathogenesis of FD and that may be a potential therapeutic mechanism of Baizhu on FD. For example, MAOA and MAOB are the widely distributed mitochondrial enzyme with high expression levels in gastro-intestinal and hepatic as well as neuronal tissues, and are genetically associated with the pathogenesis of mental disorders (Lin et al., 2000); In addition, NOS1 and NOS3 can play a role in the pathogenesis and symptom of depression, NOS2 is generally up-regulated in various tissues under inflammatory conditions (Chakrabarti et al., 2012). Moreover, SLC6A4 is significantly related with both increased depressive symptoms and elevated IL-6 plasma levels suggesting that common phathophysiological processes may be associated with depression and inflammation (Su et al., 2009). It is worthy to mention that STAT3 rs2293152 polymorphism may be associated with the occurrence of ulcerative colitis and might be used as a predictive factor for ulcerative colitis (Wang et al., 2014). Overall, these results suggested that Zhishi and Baizhu act synergistically to treat FD by regulating inflammation, gastrointestinal peristalsis, and mental disorders.

### Contribution score analysis

**Figure 4:**
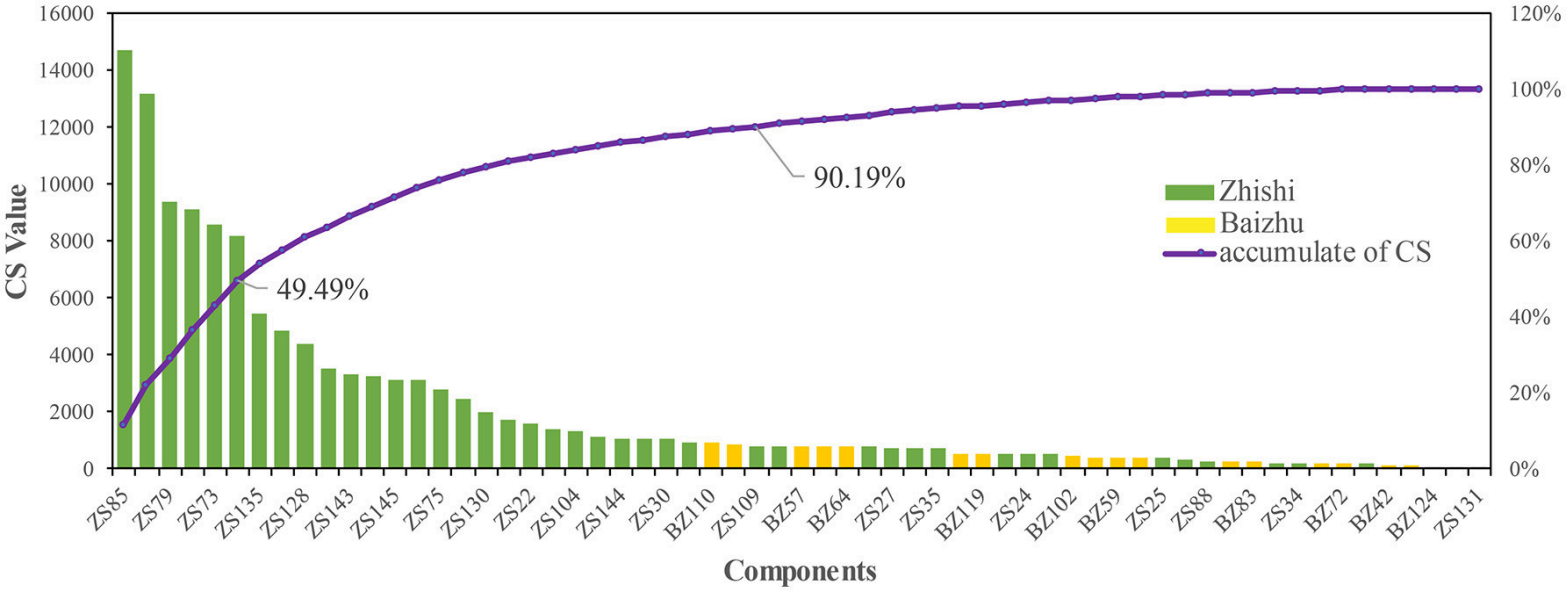
The CS and accumulative CS of active components in ZZW.

A mathematical formula was established to simulate the effect of each component of ZZW on the treatment of FD. The CS value of each active component in ZZW is calculated and showed in Figure 4 and Table S4. According to the calculation results, the top 6 components with a sum of CS of 49.49% are acacetin (ZS85), luteolin (ZS137), chrysoeriol (ZS79), 5,7,4′-Trimethylapigenin (ZS107), diosmetin (ZS73), Tetramethoxyluteolin (ZS117), and 29 components can contribute the effects of ZZW on FD with a sum of CS of 90.18%. It has been proved that the effective therapeutic effect of ZZW on FD is derived from all active components, rather than a few components. These results may fully clarify why the herbs in ZZW could generate synergistic and combination effects on FD.

### Potential synergistic mechanisms analysis of zhishi and baizhu

#### GO enrichment analysis for targets

GO enrichment analysis based on DAVID Functional Annotation Clustering Tool was performed to identify the biological significance of the primary target with FDR > 0.01 and the gene count above the mean value.

In the C-T network (Figure 3), 37 (84%) components in Zhishi and 17 (94%) components in Baizhu have 18 same targets, including MAPT, OPRD1, OPRK1, OPRL1, OPRM1, AR, PTGS1, PTGS2, DRD2, DRD3, NOS1, NOS2, NOS3, MAOA, MAOB, ACE, SRD5A1, and SLC6A2. Surprisingly, these targets are mainly distributed in GO:0042755 eating behavior (OPRD1, OPRK1, OPRL1, OPRM1), GO:0006809 nitric oxide biosynthetic process, GO:0045909 positive regulation of vasodilation (NOS1, NOS2, NOS3), GO:0019229 regulation of vasoconstriction (ACE), GO:0042420 dopamine catabolic process (MAOA, MAOB), GO:0042417 dopamine metabolic process (DRD2, DRD3), GO:0007611 learning or memory (MAPT, DRD3, PTGS2), GO:0006954 inflammatory response (PTGS1, PTGS2), GO:0042493 response to drug (SRD5A1, SLC6A2, MAOB, DRD2, DRD3, PTGS2). Ninety percent of these GO terms are located on the related GO terms of FD. These results suggest that targets are related to FD at different levels, indicating that ZZW could produce a combination effect on FD.

**Figure 5:**
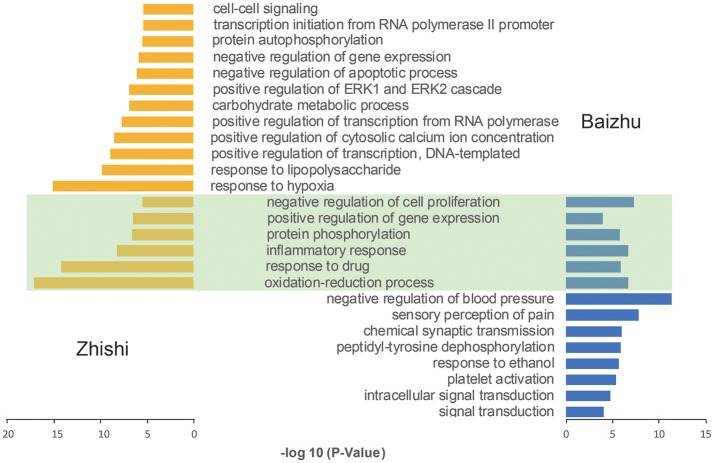
Go enrichment analysis of the targets of ZZW. The green part represents the shared GO terms of Zhishi and Baizhu.

In order to further dissect the combination effects of Zhishi and Baizhu, all the target interacting with the active components of Zhishi and Baizhu were enriched by GO enrichment analysis, respectively. As shown in Figure 5, there are six shared GO biological process (BP) terms between Zhishi and Baizhu, including oxidation-reduction process, inflammatory response, protein phosphorylation, and so on are all closely associated with FD. For instance, the oxidation-reduction process has previously been shown to correlate with the pathogenesis of depression (Grases et al., 2014) and inflammatory diseases of the gastrointestinal tract (such as *H. pylori* infection and IBD) (Van Hecke et al., 2017), and the role of inflammatory response in FD is extensive, such as anti-depression (Miller and Raison, 2016), eradicating H. pylori infection and improving dyspepsia (White et al., 2015), etc. To our surprise, 18 common gene GO terms matched only one-third of the 6 shared GO terms, this results prove once again that the treatment of ZZW for FD is a synergistic effect form.

In addition, the other 12 groups of Zhishi are also related to the treatment of FD. For instance, many investigations suggest that the regulation of cytosolic calcium ion concentration has an important role in anti-depression treatment (Yamawaki et al., 2001), and the abnormalities of ERK1/2 signaling may be crucial for the vulnerability of depression (Dwivedi and Zhang, 2016), moreover, the ERK activity constitutively or transiently may serve as a negative regulator of vascular inflammation by suppressing endothelial NF-κB activation, and play an anti-inflammatory role (Maeng et al., 2006). The other eight groups of Baizhu are also related to FD. For instance, patients with functional dyspepsia have a lower threshold both to the initial symptomatic recognition and to the perception of pain during gastric distension (Bradette et al., 1991), and depression is associated with increased platelet activation (Morel-Kopp et al., 2009).

Collectively, these results suggest that Zhishi and Baizhu may play synergistic and complementary effects on FD from the perspective of GO enrichment analysis.

#### Pathway analysis to explore the therapeutic mechanisms of ZZW

**Figure 6:**
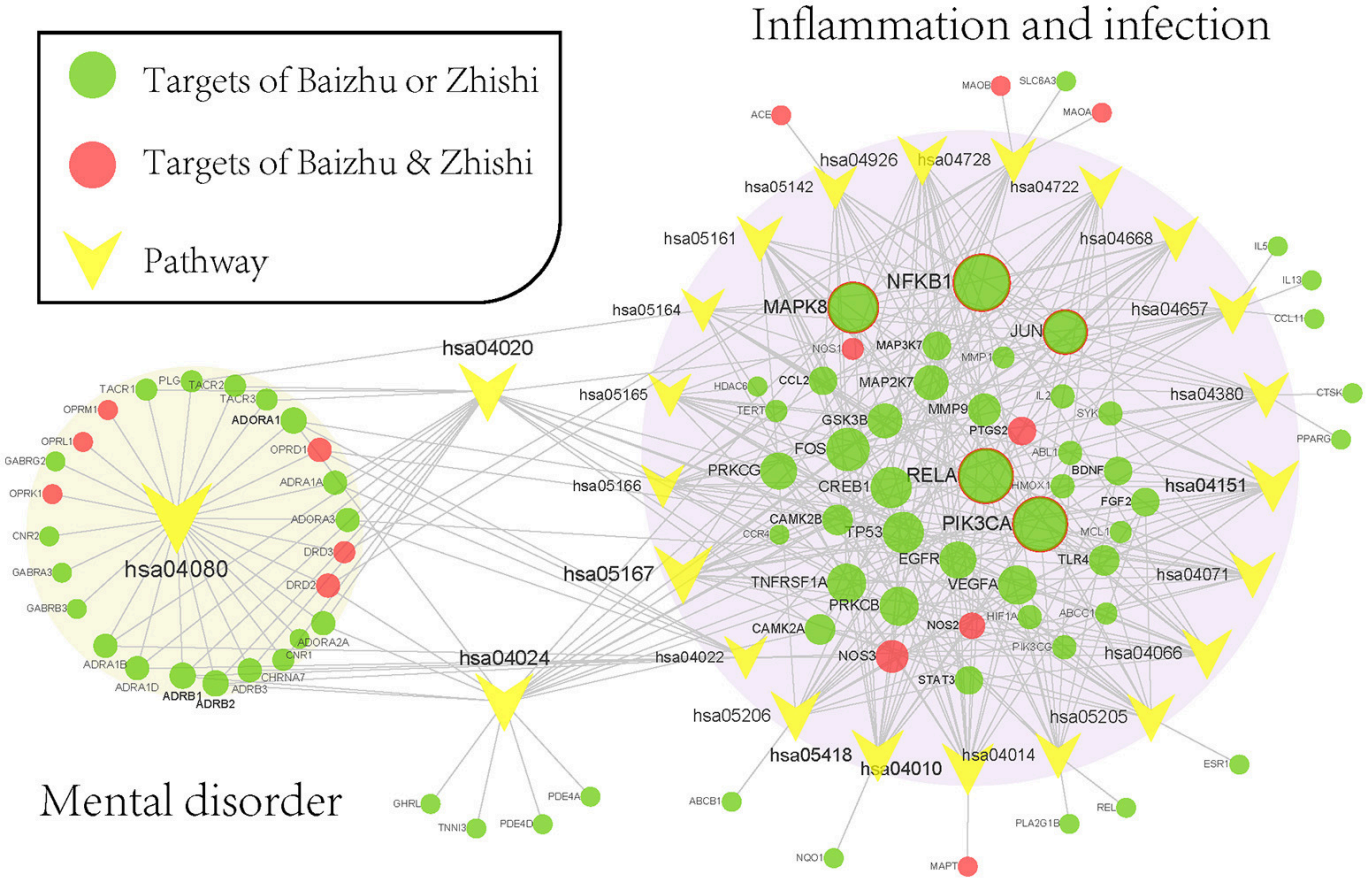
Target-pathway network of ZZW. The red nodes are the common targets of Zhishi and Baizhu, and the green represents the different targets, the yellow represents the pathways. The orange area represents the depression-related pathway, the green area represents the inflammation and infection-related pathway.

To elaborate on the significant pathways involved in ZZW for FD therapy, all target proteins were mapped onto KEGG pathways with degree ≥ 12 (the median valve) resulting in a target-pathway (T-P) network (Figure 6). The T-P network contains 108 nodes (24 pathways and 84 targets and 353 edges). NFKB1, PIK3CA, RELA, MAPK8, and JUN were in the top-ranking degrees in the T-P network and linked by 19, 18, 18, 16, and 13 pathways (Figure 6). NFKB1 encoding pro-inflammatory cytokines, chemokines, and molecules involved in carcinogenesis was markedly up-regulated in *H. pylori* GC026-challenged cells (Castaño-Rodríguez et al., 2015); PIK3CA can active the PI3K signaling pathway in gastric cancer through up-regulation or mutation (Li et al., 2005); RELA, the principal effector of canonical NF-κB signaling (Parker et al., 2014); MAPK8 was mediators of signal transduction from the cell surface to the nucleus, and can regulate AP-1 transcriptional activity by multiple mechanisms (Whitmarsh and Davis, 1996); JUN were phosphorylated through homeodomain-interacting protein kinase 3 after cAMP stimulation (Lan et al., 2007). Noticeably, the target in the top-ranking degrees were almost related to FD inducing factors, such as inflammation and organisms infection, indicating that anti-inflammation and anti-microbial play a crucial role in the treatment of FD.

The pathways associated with these targets showed more significant features (Figure 5), Neuroactive ligand-receptor interaction (hsa04080) pathway exhibits the highest number of target connections (degree = 25), followed by Calcium signaling pathway (hsa04020, degree = 19), Kaposi's sarcoma-associated herpesvirus infection (hsa05167, n = 19), cAMP signaling pathway (hsa04024, degree = 19), Fluid shear stress and atherosclerosis (hsa05418, degree = 16). Based on the results of pathways analysis, it was found that these high-degree pathways were closely related to neuroprotection, anti-inflammation, and anti-microbial. Specially, the crucial neuroactive ligand-receptor interaction pathway has been applied into the analysis of mental disorders (Adkins et al., 2012; Kong et al., 2015), which is regulated by 25 potential targets (ADORA1, ADORA2A, ADORA3, etc.). In addition, calcium signaling pathway is a major signal transduction, and can affect the development of some of the major psychiatric diseases such as bipolar disorder and schizophrenia by regulating neuronal excitability, information processing and cognition (Berridge, 2014). Nevertheless, cAMP is one of the most common and universal second messengers, and was proven that its abnormalities would be linked with psychotic depression (Perez et al., 2002).

**Figure 7:**
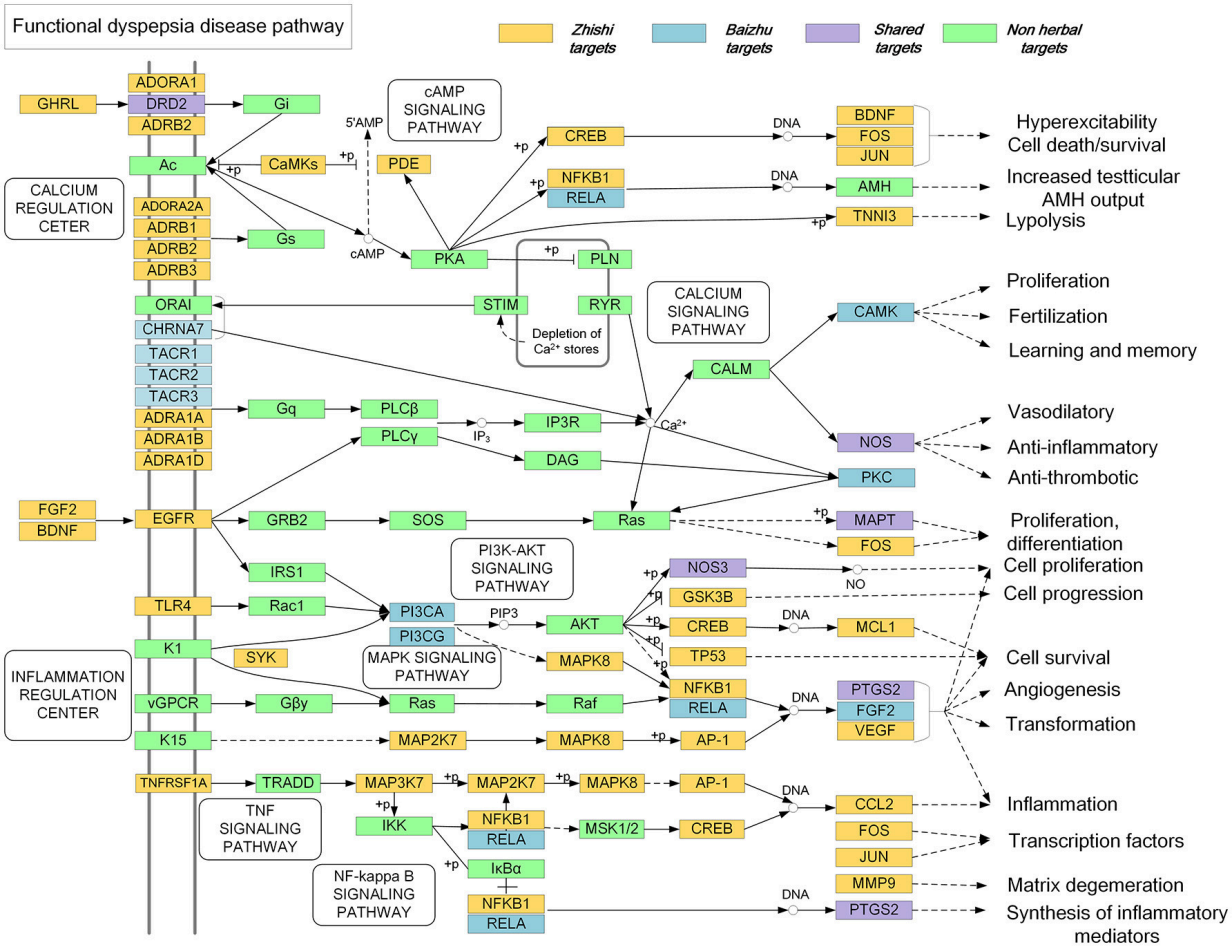
Distribution of target proteins of ZZW on the compressed FD pathway.

In order to further explore the synergetic mechanism of Zhishi and Baizhu in the treatment of FD in ZZW, we have constructed a comprehensive pathway. As shown in Figure 7, in the calcium regulation center, Zhishi can act on the genes of the upstream pathway, such as ADRA1A, ADRA1B, and ADRA1D, ADORA2A, DRD2, while Baizhu can act the genes in downstream, such as PRKCB, CAMK2A, and NOS1, these results can indicate Zhishi and Baizhu play synergistic and complementary effects on learning and memory, vasodilatory, anti-inflammatory, and anti-thrombotic.

Additionally, in the inflammation regulation center, Zhishi can act the genes of the upstream pathway, such as FGF2, BDNF, TLR4, and TNFRSF1A, while Baizhu can act the gene in the downstream pathway, such as PIK3CA, CCL2, and PTGS2, which are associated with the pathway of inflammation and synthesis of inflammatory mediators.

As the pathogenic factors of FD are related to inflammation, mental disorder, and organisms infection, so the above results suggest that Zhishi and Baizhu can exert a synergistic effect on FD at the pathway level.

## Discussion

FD is a common digestive disease associated with many pathogenic factors, such as gastric and duodenal perturbations (Tack and Talley, 2013), organisms infection (Futagami et al., 2015), mental disorders (Aro et al., 2015), etc. The related genes of FD include NFKB1 (Castaño-Rodríguez et al., 2015), PIK3CA (Li et al., 2005), RELA (Parker et al., 2014), MAPK8 (Whitmarsh and Davis, 1996), JUN (Lan et al., 2007), and etc; and the involved pathway include neuroactive ligand-receptor interaction pathway (Kong et al., 2015), calcium signaling pathway (Berridge, 2014), cAMP signaling pathway (Perez et al., 2002), MAPK signaling pathway (Allison et al., 2009), NF-κB pathway (Marengo et al., 2018), and etc. Our study found that ZZW can treat FD by adjusting the related genes and pathways of dyspepsia, *Helicobacter pylori* infection, and depression. Thus, it is indirectly confirmed the relationship between FD and the above-mentioned pathogenic factors.

In this manuscript, we illuminate the synergistic effect of ZZW on FD from four aspects. Firstly, the C-T network showed 80 percent of the components in Zhishi and Baizhu have 18 same targets, involving GO:0042755 eating behavior, GO:0006809 nitric oxide biosynthetic process, GO:0045909 positive regulation of vasodilation, GO:0019229 regulation of vasoconstriction, GO:0042420 dopamine catabolic process, GO:0042417 dopamine metabolic process, GO:0007611 learning or memory, GO:0006954 inflammatory response, and GO:0042493 response to drug. This indicates that the herbs in ZZW have the cooperation effects on FD. Secondly, the CS of each component in ZZW are calculated and showed that 29 components can contribute the effects of ZZW for FD with a sum of 90.18% of CS. It is proved that the effective therapeutic effect of ZZW on FD is derived from all active components, not a few components. Thirdly, GO enrichment analysis indicated that all the target interacting with the active components of Zhishi and Baizhu have six shared GO BP terms, which are all closely associated with FD, whereas the 18 same targets GO terms cannot cover the shared GO terms of the target interacting with the all components, and the other components also have action, namely the components work together to play a synergistic effect. Finally, the pathway analysis proves again that Zhishi and Baizhu can exert a synergistic effect on the treatment of FD through acting the upstream and downstream gene in the calcium signaling pathway, cAMP signaling pathway, MAPK signaling pathway, and NF-κB pathway. Recent studies also established that the compatibility of Zhishi and Baizhu can promote the function of modulation of gastroinfestinal motility via regulating the levels of MTL and VIP (Li et al., 2007). All these results suggest that ZZW could produce a combination effect on FD.

In this study, system pharmacology and network pharmacology were used to construct a strategy for decoding the TCM pharmacologic molecular mechanism. This strategy combined physicochemical properties, network topological features, function analysis, and pathway analysis, and provided a reference for the new methods.

Currently, system pharmacology provides a powerful tool for exploring the compatibility and mechanism of TCM formulae (Yue et al., 2017), but its findings mainly rely on theoretical analyses, thus additional experiments are needed to validate our findings as well as potential clinical significance. It is noteworthy that the OB values of four flavanone glycoside which are the high content in Zhishi (Zeng et al., 2016), were <30%. Therefore, the metabolites of these flavanone glycosides by gut microbiota may be a critical step in the emergence of their bioactivities *in vivo*, especially under the disease state (Chen F. et al., 2016).

## Author contributions

A-PL, Z-LL, and D-GG provided the concept and designed the study. CW, QR, and X-TC conducted the analyses and wrote the manuscript. CW, QR, X-TC, Z-QS, Z-CN, J-HG, X-LM, and D-RL participated in data analysis. A-PL, Z-LL, and D-GG contributed to revising and proof-reading the manuscript. All authors read and approved the final manuscript.

### Conflict of interest statement

The authors declare that the research was conducted in the absence of any commercial or financial relationships that could be construed as a potential conflict of interest. The reviewer CF and handling Editor declared their shared affiliation.
