# Supplementary material for: System Pharmacology-Based Strategy to Decode the Synergistic Mechanism of Zhi-zhu Wan for Functional Dyspepsia
Source: Front Pharmacol. 2018 Aug 6;9:841. doi: 10.3389/fphar.2018.00841 (PMC6087764; doi:10.3389/fphar.2018.00841)
Supplement: Supplementary file 1 [file Table_1.DOCX]

**Table S1** The detail information of the components in Zhishi and Baizhu

| **ID** | **molecule_name** | **MW** | **OB** | **CACO2** | **DL** | **MLOGP** | **H-bond acceptors** | **H-bond donors** | **TPSA** | **GI absorption** |
| --- | --- | --- | --- | --- | --- | --- | --- | --- | --- | --- |
| BZ1 | serine | 105.09 | 109.16 | -1.23 | 0.01 | -3.91 | 4.00 | 3.00 | 83.55 | High |
| BZ2 | glutamic acid | 147.13 | 104.46 | -1.14 | 0.02 | -3.18 | 5.00 | 3.00 | 100.62 | High |
| BZ3 | 3,3,7,11-tetramethyl-tricyclo[6 | 204.36 | 53.28 | 1.83 | 0.10 | 5.65 | 0.00 | 0.00 | 0.00 | Low |
| BZ4 | 2alpha-(alpha-Methylbutyrul)-oxy-5alpha,7belta,9alpha,10belta-tetraacetoxy-4(20),11-taxadiene | 604.74 | 23.74 | -0.42 | 0.78 | 3.57 | 10.00 | 0.00 | 131.50 | Low |
| BZ5 | eudesma-4(14),11-diene | 204.36 | 17.37 | 1.85 | 0.08 | 4.63 | 0.00 | 0.00 | 0.00 | Low |
| BZ6 | histidine | 155.16 | 56.03 | -0.52 | 0.03 | -3.74 | 4.00 | 3.00 | 92.00 | High |
| BZ7 | daphnetin | 178.14 | 23.84 | 0.43 | 0.07 | 0.45 | 4.00 | 2.00 | 70.67 | High |
| BZ8 | Apigenin 8-C-glucoside | 432.38 | 9.94 | -1.26 | 0.71 | -2.02 | 10.00 | 7.00 | 181.05 | Low |
| BZ9 | 4-styrylpyridazine | 182.23 | 18.32 | 1.34 | 0.06 | 2.22 | 2.00 | 0.00 | 25.78 | High |
| BZ10 | phenol | 94.11 | 36.05 | 1.46 | 0.01 | 1.45 | 1.00 | 1.00 | 20.23 | High |
| BZ11 | 4H-1-Benzopyran-4-one | 146.15 | 31.22 | 1.06 | 0.04 | 0.84 | 2.00 | 0.00 | 30.21 | High |
| BZ12 | icariside F2 | 402.40 | 5.67 | -1.43 | 0.47 | -2.43 | 10.00 | 6.00 | 158.30 | Low |
| BZ13 | proline | 115.13 | 55.46 | 0.17 | 0.01 | -2.59 | 3.00 | 2.00 | 49.33 | High |
| BZ14 | β-elemol | 222.37 | 30.90 | 1.36 | 0.07 | 3.56 | 1.00 | 1.00 | 20.23 | High |
| BZ15 | β-farnesene | 204.36 | 19.02 | 1.96 | 0.05 | 4.84 | 0.00 | 0.00 | 0.00 | Low |
| BZ16 | Squalene | 410.73 | 33.54 | 2.10 | 0.42 | 7.93 | 0.00 | 0.00 | 0.00 | Low |
| BZ17 | β-Amyrin acetate | 468.77 | 42.06 | 1.36 | 0.74 | 7.08 | 2.00 | 0.00 | 26.30 | Low |
| BZ18 | taraxeryl acetate | 468.77 | 9.91 | 1.39 | 0.74 | 7.08 | 2.00 | 0.00 | 26.30 | Low |
| BZ19 | spathulenol | 220.36 | 81.65 | 1.45 | 0.12 | 3.67 | 1.00 | 1.00 | 20.23 | High |
| BZ20 | atractyloside A | 448.51 | 2.78 | -1.73 | 0.57 | -1.95 | 10.00 | 7.00 | 177.14 | Low |
| BZ21 | atractyloside B | 450.53 | 1.46 | -1.65 | 0.57 | -1.80 | 10.00 | 8.00 | 180.30 | Low |
| BZ22 | longifolene-(V4) | 204.36 | 48.74 | 1.78 | 0.11 | 5.65 | 0.00 | 0.00 | 0.00 | Low |
| BZ23 | Cis-(-)-2,4a,5,6,9a-hexahydro-3,5,5,9-tetramethyl-(1H)benzocycloheptene | 204.36 | 50.61 | 1.84 | 0.08 | 4.63 | 0.00 | 0.00 | 0.00 | Low |
| BZ24 | 1,2,3,6-tetramethyl-bicyclo[2 | 162.28 | 45.43 | 1.84 | 0.06 | 3.84 | 0.00 | 0.00 | 0.00 | Low |
| BZ25 | 2-isopropenyl-4a,8-dimethyl-1,2,3,4,4a,5,6,7-octahydronaphthalene | 204.36 | 27.54 | 1.82 | 0.08 | 4.63 | 0.00 | 0.00 | 0.00 | Low |
| BZ26 | delta-selinene | 204.36 | 20.92 | 1.85 | 0.08 | 4.63 | 0.00 | 0.00 | 0.00 | Low |
| BZ27 | atractylenolactam | 229.32 | 56.48 | 1.23 | 0.15 | 2.85 | 1.00 | 1.00 | 29.10 | High |
| BZ28 | biepiasterolide | 462.63 | 10.68 | 0.84 | 0.81 | 5.43 | 4.00 | 0.00 | 52.60 | High |
| BZ29 | 2-methoxy-4-methyl-phenol | 138.17 | 28.23 | 1.35 | 0.03 | 1.48 | 2.00 | 1.00 | 29.46 | High |
| BZ30 | m-cresol | 108.14 | 53.95 | 1.50 | 0.01 | 1.81 | 1.00 | 1.00 | 20.23 | High |
| BZ31 | α-curcumene | 202.34 | 28.12 | 1.93 | 0.06 | 5.75 | 0.00 | 0.00 | 0.00 | Low |
| BZ32 | o-cresol | 108.14 | 61.87 | 1.54 | 0.02 | 1.81 | 1.00 | 1.00 | 20.23 | High |
| BZ33 | β-humulene | 204.36 | 22.93 | 1.86 | 0.06 | 4.53 | 0.00 | 0.00 | 0.00 | Low |
| BZ34 | α-Caryophyllene | 204.36 | 25.25 | 1.86 | 0.06 | 4.53 | 0.00 | 0.00 | 0.00 | Low |
| BZ35 | α-humulene | 204.36 | 25.25 | 1.86 | 0.06 | 4.53 | 0.00 | 0.00 | 0.00 | Low |
| BZ36 | α-bisabolol | 222.37 | 17.19 | 1.40 | 0.07 | 3.56 | 1.00 | 1.00 | 20.23 | High |
| BZ37 | humulen-(v1) | 204.36 | 27.77 | 1.87 | 0.09 | 4.63 | 0.00 | 0.00 | 0.00 | Low |
| BZ38 | caryophyllene | 204.36 | 25.05 | 1.84 | 0.09 | 4.63 | 0.00 | 0.00 | 0.00 | Low |
| BZ39 | isocaryophyllene | 204.36 | 25.05 | 1.84 | 0.09 | 4.63 | 0.00 | 0.00 | 0.00 | Low |
| BZ40 | β-eudesmol | 222.37 | 25.08 | 1.29 | 0.10 | 3.67 | 1.00 | 1.00 | 20.23 | High |
| BZ41 | Jurubine | 595.82 | 3.10 | -1.27 | 0.42 | 1.78 | 9.00 | 6.00 | 154.86 | Low |
| BZ42 | Anhydroatractylolide | 234.34 | 52.24 | 1.24 | 0.15 | 3.44 | 2.00 | 0.00 | 26.30 | High |
| BZ43 | longifolene-I2 | 204.36 | 49.25 | 1.83 | 0.11 | 5.65 | 0.00 | 0.00 | 0.00 | Low |
| BZ44 | β-vatirenene | 202.34 | 29.62 | 1.87 | 0.08 | 4.53 | 0.00 | 0.00 | 0.00 | Low |
| BZ45 | seychellene | 204.36 | 16.97 | 1.82 | 0.12 | 5.65 | 0.00 | 0.00 | 0.00 | Low |
| BZ46 | patchoulene | 204.36 | 51.99 | 1.81 | 0.11 | 5.65 | 0.00 | 0.00 | 0.00 | Low |
| BZ47 | isoledene | 204.36 | 54.51 | 1.85 | 0.10 | 5.65 | 0.00 | 0.00 | 0.00 | Low |
| BZ48 | cedrene | 204.36 | 51.26 | 1.81 | 0.10 | 5.65 | 0.00 | 0.00 | 0.00 | Low |
| BZ49 | alloaromadendrene | 204.36 | 50.47 | 1.82 | 0.10 | 5.65 | 0.00 | 0.00 | 0.00 | Low |
| BZ50 | cyperene | 204.36 | 53.34 | 1.82 | 0.11 | 5.65 | 0.00 | 0.00 | 0.00 | Low |
| BZ51 | β-sitosterol | 414.72 | 53.34 | 1.82 | 0.11 | 6.73 | 1.00 | 1.00 | 20.23 | Low |
| BZ52 | 4-Ethoxycarbonyl-2-quinolone | 217.22 | 66.97 | 0.44 | 0.10 | 1.80 | 3.00 | 1.00 | 59.16 | High |
| BZ53 | 6, 7-dimethoxycoumarin | 206.20 | 66.97 | 0.44 | 0.10 | 1.05 | 4.00 | 0.00 | 48.67 | High |
| BZ54 | Scopoletin 7-O-beta-D-Xylopyranosyl-(1→6)-beta-D-glucopyranoside | 486.43 | 4.89 | -1.98 | 0.83 | -2.70 | 13.00 | 6.00 | 197.74 | Low |
| BZ55 | Syringin | 372.37 | 26.80 | -1.01 | 0.33 | -1.59 | 9.00 | 5.00 | 138.07 | Low |
| BZ56 | 6-Isopropenyl-4,8a-dimethyl-1,2,3,5,6,7,8,8a-octahydro-naphthalen-2-ol | 220.36 | 10.38 | -0.96 | 0.33 | 3.56 | 1.00 | 1.00 | 20.23 | High |
| BZ57 | 8β-methoxy-atractylenolide I | 260.33 | 54.47 | 1.02 | 0.19 | 2.63 | 3.00 | 0.00 | 35.53 | High |
| BZ58 | Atracty lentrid | 232.28 | 16.62 | -0.06 | 0.09 | 1.62 | 3.00 | 3.00 | 60.69 | High |
| BZ59 | 14α-methyl butyryl-14-acetyl-2E,8E,10E-atractylentriol | 316.40 | 64.50 | 0.20 | 0.23 | 2.71 | 4.00 | 2.00 | 66.76 | High |
| BZ60 | 12α-methylbutyryl-14-acetyl-2E,8Z,10E-atractylentriol | 358.43 | 62.69 | 0.41 | 0.29 | 3.07 | 5.00 | 1.00 | 72.83 | High |
| BZ61 | 14α-methyl butyryl-14-acetyl-2E,8Z,10E-atractylentriol | 316.40 | 29.83 | 0.04 | 0.23 | 2.71 | 4.00 | 2.00 | 66.76 | High |
| BZ62 | 2,3-dimethyl-phenol | 122.17 | 53.02 | 1.59 | 0.02 | 2.14 | 1.00 | 1.00 | 20.23 | High |
| BZ63 | Scutellarein-6-O-glucoside | 448.38 | 7.19 | -1.23 | 0.77 | -2.10 | 11.00 | 7.00 | 190.28 | Low |
| BZ64 | 8β-ethoxy atractylenolide- II | 276.38 | 56.48 | 1.08 | 0.21 | 3.37 | 3.00 | 0.00 | 35.53 | High |
| BZ65 | icarisideD1 | 416.42 | 5.70 | -1.46 | 0.52 | -2.20 | 10.00 | 6.00 | 158.30 | Low |
| BZ66 | atractylenolide IV | 306.36 | 16.20 | 0.30 | 0.28 | 2.41 | 5.00 | 1.00 | 72.83 | High |
| BZ67 | 6-( β-D-glucopyranosyloxy ) -5-hydroxy-7-methoxy-2- phenyl-4H-1-Benzopyran-4-one | 446.41 | 9.11 | -0.86 | 0.75 | -1.39 | 10.00 | 5.00 | 159.05 | Low |
| BZ68 | dihydrosyrindine | 374.39 | 18.33 | -0.85 | 0.32 | -1.50 | 9.00 | 5.00 | 138.07 | Low |
| BZ69 | 10-epiatractyloside A | 448.51 | 3.31 | -2.00 | 0.57 | -1.95 | 10.00 | 7.00 | 177.14 | Low |
| BZ70 | (2E)-2-decene- 4,6-diyne-1,8-diol 8-O-b-D-apiofuranosyl-(1→6)-b-D-glucopyranoside | 458.46 | 7.58 | -1.57 | 0.58 | -2.51 | 11.00 | 7.00 | 178.53 | Low |
| BZ71 | Stereolensin | 464.38 | 5.74 | -1.43 | 0.80 | -2.59 | 12.00 | 8.00 | 210.51 | Low |
| BZ72 | Isoasterolide A | 232.32 | 52.65 | 1.27 | 0.15 | 3.35 | 2.00 | 0.00 | 26.30 | High |
| BZ73 | atractylenolide V | 264.32 | 16.37 | 0.23 | 0.20 | 2.02 | 4.00 | 2.00 | 66.76 | High |
| BZ74 | atractylenolide VI | 202.34 | 20.97 | 1.85 | 0.08 | 4.53 | 0.00 | 0.00 | 0.00 | Low |
| BZ75 | atractylenolide VII | 262.39 | 40.99 | 1.32 | 0.14 | 3.84 | 2.00 | 0.00 | 26.30 | High |
| BZ76 | 2-Propenoic acid，3-( 3，4-dihydroxyphenyl)-，4-( 6-β-D-glucopyranosyl-5，7-dihydroxy-4-oxo-4H-1-benzopyran-2-yl) -2-hydroxyphenyl ester，( 2E) | 610.52 | 2.96 | -1.78 | 0.58 | -1.92 | 14.00 | 9.00 | 247.81 | Low |
| BZ77 | Methose | 180.16 | 2.02 | -1.82 | 0.03 | -2.91 | 6.00 | 5.00 | 118.22 | Low |
| BZ78 | Polymannose | 180.16 | 25.33 | -1.83 | 0.03 | -2.91 | 6.00 | 5.00 | 118.22 | Low |
| BZ79 | LPG | 89.09 | 87.62 | -0.27 | 0.01 | -4.53 | 2.00 | 1.00 | 67.77 | Low |
| BZ80 | Gulutamine | 146.15 | 88.36 | -1.01 | 0.02 | -3.58 | 4.00 | 3.00 | 106.41 | High |
| BZ81 | stigmast-22E-en-3beta-ol | 414.72 | 6.65 | 1.36 | 0.76 | 6.73 | 1.00 | 1.00 | 20.23 | Low |
| BZ82 | uridine | 244.20 | 19.97 | -1.29 | 0.11 | -2.24 | 6.00 | 4.00 | 124.78 | Low |
| BZ83 | atractylodes macrocephala | 462.68 | 45.96 | 0.85 | 0.81 | 2.47 | 3.00 | 1.00 | 46.53 | High |
| BZ84 | biatractylolide | 462.63 | 45.96 | 0.84 | 0.81 | 5.43 | 4.00 | 0.00 | 52.60 | High |
| BZ85 | 2-[(1R,3S,4S)-3-isopropenyl-4-methyl-4-vinylcyclohexyl]propan-2-ol | 222.37 | 30.90 | 1.36 | 0.07 | 3.56 | 1.00 | 1.00 | 20.23 | High |
| BZ86 | palmitic acid | 256.43 | 19.30 | 1.10 | 0.10 | 4.19 | 2.00 | 1.00 | 37.30 | High |
| BZ87 | (3S)-3-[(1R)-1,5-dimethylhex-4-enyl]-6-methylenecyclohexene | 204.36 | 25.11 | 1.93 | 0.06 | 4.53 | 0.00 | 0.00 | 0.00 | Low |
| BZ88 | alpha-humulene | 204.36 | 25.25 | 1.91 | 0.06 | 4.53 | 0.00 | 0.00 | 0.00 | Low |
| BZ89 | γ-elemene | 204.36 | 30.76 | 1.92 | 0.06 | 4.53 | 0.00 | 0.00 | 0.00 | Low |
| BZ90 | β-Selinene | 204.36 | 24.43 | 1.84 | 0.08 | 4.63 | 0.00 | 0.00 | 0.00 | Low |
| BZ91 | D-Serin | 105.09 | 102.86 | -0.96 | 0.01 | -3.91 | 4.00 | 3.00 | 83.55 | High |
| BZ92 | ASI | 133.10 | 36.77 | -0.99 | 0.02 | -5.05 | 4.00 | 2.00 | 105.07 | Low |
| BZ93 | Scopoletol | 192.17 | 27.77 | 0.70 | 0.08 | 0.76 | 4.00 | 1.00 | 59.67 | High |
| BZ94 | L-Lysin | 146.19 | 71.14 | -0.91 | 0.02 | -2.67 | 4.00 | 3.00 | 89.34 | High |
| BZ95 | β-caryophyllene | 204.36 | 26.64 | 1.85 | 0.09 | 4.63 | 0.00 | 0.00 | 0.00 | Low |
| BZ96 | selina-4(14),7(11)-dien-8-one | 218.34 | 32.57 | 1.40 | 0.10 | 3.46 | 1.00 | 0.00 | 17.07 | High |
| BZ97 | juniper camphor | 222.37 | 33.21 | 1.31 | 0.10 | 3.67 | 1.00 | 1.00 | 20.23 | High |
| BZ98 | Akridin | 179.22 | 33.71 | 1.62 | 0.10 | 3.06 | 1.00 | 0.00 | 12.89 | High |
| BZ99 | D-Camphene | 136.24 | 36.60 | 1.81 | 0.04 | 4.29 | 0.00 | 0.00 | 0.00 | Low |
| BZ100 | 8β-ethoxy atractylenolide Ⅲ | 276.38 | 56.48 | 1.08 | 0.21 | 3.37 | 3.00 | 0.00 | 35.53 | High |
| BZ101 | (3S,8S,9S,10R,13R,14S,17R)-10,13-dimethyl-17-[(2R,5S)-5-propan-2-yloctan-2-yl]-2,3,4,7,8,9,11,12,14,15,16,17-dodecahydro-1H-cyclopenta[a]phenanthren-3-ol | 428.75 | 36.23 | 1.30 | 0.79 | 6.92 | 1.00 | 1.00 | 20.23 | Low |
| BZ102 | atractylenolide I | 230.31 | 35.21 | 1.32 | 0.15 | 3.26 | 2.00 | 0.00 | 26.30 | High |
| BZ103 | 2-[(2R,5S,6S)-6,10-dimethylspiro[4.5]dec-9-en-2-yl]propan-2-ol | 222.37 | 15.49 | 1.43 | 0.09 | 3.67 | 1.00 | 1.00 | 20.23 | High |
| BZ104 | α-Amyrin | 426.73 | 9.98 | 1.38 | 0.76 | 6.92 | 1.00 | 1.00 | 20.23 | Low |
| BZ105 | Hemo-sol | 136.24 | 39.49 | 1.85 | 0.02 | 3.27 | 0.00 | 0.00 | 0.00 | Low |
| BZ106 | Ethyl pivaloylacetate | 172.22 | 47.60 | 0.80 | 0.03 | 1.29 | 3.00 | 0.00 | 43.37 | High |
| BZ107 | atractylone | 216.32 | 26.00 | 1.74 | 0.13 | 3.42 | 1.00 | 0.00 | 13.14 | High |
| BZ108 | PHA | 165.19 | 71.45 | 0.31 | 0.04 | -1.11 | 3.00 | 2.00 | 63.32 | High |
| BZ109 | (5E,9Z)-3,6,10-trimethyl-4,7,8,11-tetrahydrocyclodeca[b]furan | 216.32 | 45.41 | 1.77 | 0.10 | 3.33 | 1.00 | 0.00 | 13.14 | High |
| BZ110 | atractylenolideII | 232.32 | 43.54 | 1.31 | 0.15 | 3.35 | 2.00 | 0.00 | 26.30 | High |
| BZ111 | arginine | 174.20 | 57.95 | -0.64 | 0.03 | -3.21 | 3.00 | 4.00 | 132.16 | Low |
| BZ112 | GLY | 75.07 | 51.32 | -0.52 | 0.00 | -5.05 | 2.00 | 1.00 | 67.77 | Low |
| BZ113 | DIBP | 278.35 | 20.97 | 0.91 | 0.13 | 3.43 | 4.00 | 0.00 | 52.60 | High |
| BZ114 | (1S,2R,4R)-Neoiso-dihydrocarveol | 154.25 | 50.50 | 1.20 | 0.03 | 2.30 | 1.00 | 1.00 | 20.23 | High |
| BZ115 | Istidina | 155.16 | 56.03 | -0.52 | 0.03 | -3.74 | 4.00 | 3.00 | 92.00 | High |
| BZ116 | α-Longipinene | 204.36 | 52.13 | 1.81 | 0.12 | 5.65 | 0.00 | 0.00 | 0.00 | Low |
| BZ117 | L-Valin | 117.15 | 50.23 | 0.05 | 0.01 | -2.20 | 3.00 | 2.00 | 63.32 | High |
| BZ118 | alloaromadedrene | 204.36 | 54.11 | 1.81 | 0.10 | 5.65 | 0.00 | 0.00 | 0.00 | Low |
| BZ119 | 3β-acetoxyatractylone | 274.36 | 34.74 | 1.19 | 0.22 | 2.83 | 3.00 | 0.00 | 39.44 | High |
| BZ120 | DTY | 181.19 | 61.44 | -0.06 | 0.05 | -1.70 | 4.00 | 3.00 | 83.55 | High |
| BZ121 | isoleucine | 131.18 | 48.51 | 0.15 | 0.01 | -3.29 | 2.00 | 1.00 | 67.77 | High |
| BZ122 | 14-acetyl-12-senecioyl-2E,8E,10E-atractylentriol | 356.42 | 28.80 | 0.35 | 0.30 | 2.99 | 5.00 | 1.00 | 72.83 | High |
| BZ123 | 12-senecioyl-2E,8E,10E-atractylentriol | 314.38 | 20.41 | 0.12 | 0.21 | 2.63 | 4.00 | 2.00 | 66.76 | High |
| BZ124 | 14-acetyl-12-senecioyl-2E,8Z,10E-atractylentriol | 356.42 | 63.37 | 0.26 | 0.30 | 2.99 | 5.00 | 1.00 | 72.83 | High |
| BZ125 | atractylenolide III | 248.32 | 67.29 | 0.76 | 0.17 | 2.47 | 3.00 | 1.00 | 46.53 | High |
| BZ126 | (1R)-2-methyl-1-phenylprop-2-en-1-ol | 148.21 | 81.20 | 1.29 | 0.03 | 2.40 | 1.00 | 1.00 | 20.23 | High |
| BZ127 | Prolinum | 115.13 | 78.51 | 0.21 | 0.01 | -2.59 | 3.00 | 2.00 | 49.33 | High |
| BZ128 | (+/-)-Isoborneol | 154.25 | 83.63 | 1.20 | 0.05 | 2.45 | 1.00 | 1.00 | 20.23 | High |
| ZS1 | deacetyl nomilin | 472.53 | 25.90 | -0.39 | 0.74 | 1.45 | 8.00 | 1.00 | 115.57 | High |
| ZS2 | Rhombifoline | 244.33 | 44.56 | 1.20 | 0.17 | 2.29 | 2.00 | 0.00 | 25.24 | High |
| ZS3 | Myricetin | 318.24 | 9.97 | -0.18 | 0.31 | -1.08 | 8.00 | 6.00 | 151.59 | Low |
| ZS4 | Quercetin | 302.24 | 10.64 | 0.02 | 0.28 | -0.56 | 7.00 | 5.00 | 131.36 | High |
| ZS5 | Cyanidin-3-O-glucoside | 449.38 | 4.66 | -1.36 | 0.75 | -1.76 | 11.00 | 8.00 | 193.44 | Low |
| ZS6 | Luteolin-4'-O-glucoside | 448.38 | 36.94 | -1.24 | 0.79 | -2.10 | 11.00 | 7.00 | 190.28 | Low |
| ZS7 | Cynaroside | 448.38 | 2.68 | -1.28 | 0.78 | -2.10 | 11.00 | 7.00 | 190.28 | Low |
| ZS8 | Orientin | 448.38 | 18.30 | -1.52 | 0.75 | -2.51 | 11.00 | 8.00 | 201.28 | Low |
| ZS9 | Luteolin-3',7-di-O-glucoside | 610.52 | 13.42 | -2.80 | 0.62 | -4.16 | 16.00 | 10.00 | 269.43 | Low |
| ZS10 | Saponarin | 594.52 | 3.72 | -2.75 | 0.77 | -4.10 | 15.00 | 10.00 | 260.20 | Low |
| ZS11 | Apigetrin | 432.38 | 5.52 | -1.20 | 0.74 | -1.61 | 10.00 | 6.00 | 170.05 | Low |
| ZS12 | Amentoflavone | 538.46 | 4.58 | -0.33 | 0.65 | 0.25 | 10.00 | 6.00 | 181.80 | Low |
| ZS13 | Vitexin | 432.38 | 9.94 | -1.26 | 0.71 | -2.02 | 10.00 | 7.00 | 181.05 | Low |
| ZS14 | Umbelliferone | 162.14 | 27.37 | 0.71 | 0.05 | 1.04 | 3.00 | 1.00 | 50.44 | High |
| ZS15 | Xanthotoxol | 202.16 | 33.48 | 0.78 | 0.12 | 0.89 | 4.00 | 1.00 | 63.58 | High |
| ZS16 | Bergaptol | 202.16 | 45.16 | 0.67 | 0.12 | 0.89 | 4.00 | 1.00 | 63.58 | High |
| ZS17 | Psoralen | 186.16 | 19.60 | 1.05 | 0.10 | 1.48 | 3.00 | 0.00 | 43.35 | High |
| ZS18 | Apiin | 564.49 | 6.28 | -2.23 | 0.80 | -3.16 | 14.00 | 8.00 | 228.97 | Low |
| ZS19 | Pyracanthoside | 450.39 | 2.67 | -1.27 | 0.78 | -1.91 | 11.00 | 7.00 | 186.37 | Low |
| ZS20 | Osthol | 244.29 | 28.61 | 1.21 | 0.13 | 2.63 | 3.00 | 0.00 | 39.44 | High |
| ZS21 | 8-geranyloxypsoralen | 338.40 | 41.92 | 1.18 | 0.42 | 3.23 | 4.00 | 0.00 | 52.58 | High |
| ZS22 | 5-Geranyloxy-7-Methoxycoumarin | 328.40 | 44.23 | 1.12 | 0.30 | 3.16 | 4.00 | 0.00 | 48.67 | High |
| ZS23 | Bergamottin | 338.40 | 41.73 | 1.16 | 0.42 | 3.23 | 4.00 | 0.00 | 52.58 | High |
| ZS24 | Phellopterin | 300.31 | 37.43 | 0.98 | 0.28 | 1.82 | 5.00 | 0.00 | 61.81 | High |
| ZS25 | Isoimperatorin | 270.28 | 47.54 | 1.06 | 0.23 | 2.14 | 4.00 | 0.00 | 52.58 | High |
| ZS26 | 6'-7'-dihydroxybergamottin | 372.41 | 70.77 | 0.12 | 0.52 | 1.66 | 6.00 | 2.00 | 93.04 | High |
| ZS27 | Epoxybergamottin | 354.40 | 57.25 | 0.92 | 0.52 | 2.48 | 5.00 | 0.00 | 65.11 | High |
| ZS28 | Cnidilin | 300.31 | 42.42 | 0.95 | 0.28 | 1.82 | 5.00 | 0.00 | 61.81 | High |
| ZS29 | Cnidicin | 354.40 | 22.73 | 1.10 | 0.42 | 2.67 | 5.00 | 0.00 | 61.81 | High |
| ZS30 | Epoxyaurapten | 314.38 | 62.78 | 0.95 | 0.31 | 2.74 | 4.00 | 0.00 | 51.97 | High |
| ZS31 | nomilinic acid | 532.58 | 12.03 | -0.56 | 0.79 | 1.03 | 10.00 | 2.00 | 152.87 | Low |
| ZS32 | Nomilin glycoside | 694.72 | 61.51 | -2.03 | 0.54 | -1.17 | 15.00 | 5.00 | 232.02 | Low |
| ZS33 | nomilin | 514.56 | 14.64 | -0.38 | 0.67 | 1.80 | 9.00 | 0.00 | 121.64 | High |
| ZS34 | Byakangelicin | 334.32 | 34.89 | -0.01 | 0.35 | 0.29 | 7.00 | 2.00 | 102.27 | High |
| ZS35 | Heraclenol | 304.29 | 72.63 | 0.08 | 0.29 | 0.57 | 6.00 | 2.00 | 93.04 | High |
| ZS36 | Oxypeucedanin hydrate | 304.29 | 33.07 | -0.06 | 0.29 | 0.57 | 6.00 | 2.00 | 93.04 | High |
| ZS37 | 19-hydroxydeacetylnomilinic acid-17-β-D-glucoside | 686.70 | 9.84 | -2.72 | 0.61 | -3.00 | 16.00 | 9.00 | 277.41 | Low |
| ZS38 | Neohopadiene | 408.70 | 7.17 | 1.83 | 0.77 | 7.89 | 0.00 | 0.00 | 0.00 | Low |
| ZS39 | isoponcimarin | 330.37 | 63.28 | 0.53 | 0.31 | 1.91 | 5.00 | 0.00 | 69.04 | High |
| ZS40 | poncimarin | 330.37 | 79.20 | 0.75 | 0.35 | 1.99 | 5.00 | 0.00 | 64.50 | High |
| ZS41 | Byakangelicol | 316.31 | 45.21 | 0.76 | 0.36 | 1.08 | 6.00 | 0.00 | 74.34 | High |
| ZS42 | Oxypeucedanin | 286.28 | 66.18 | 0.87 | 0.30 | 1.39 | 5.00 | 0.00 | 65.11 | High |
| ZS43 | obacunone | 454.51 | 17.75 | -0.08 | 0.77 | 2.16 | 7.00 | 0.00 | 95.34 | High |
| ZS44 | isolimonic acid | 488.53 | 22.46 | -0.65 | 0.76 | 0.67 | 9.00 | 2.00 | 135.80 | High |
| ZS45 | limonin | 470.51 | 27.87 | -0.07 | 0.57 | 1.45 | 8.00 | 0.00 | 104.57 | High |
| ZS46 | β-pinene | 136.23 | 44.84 | 1.81 | 0.05 | 4.29 | 0.00 | 0.00 | 0.00 | Low |
| ZS47 | Abscisic acid | 264.32 | 35.63 | -0.01 | 0.13 | 1.44 | 4.00 | 2.00 | 74.60 | High |
| ZS48 | vomifoliol-9-O-β-D-glucopyranoside | 386.44 | 22.97 | -1.29 | 0.36 | -1.06 | 8.00 | 5.00 | 136.68 | Low |
| ZS49 | γ-terpinene | 136.23 | 34.67 | 1.84 | 0.02 | 3.27 | 0.00 | 0.00 | 0.00 | Low |
| ZS50 | α-terpineol-8β-D-glucopyranoside | 316.39 | 22.73 | -0.42 | 0.19 | 0.02 | 6.00 | 4.00 | 99.38 | High |
| ZS51 | α-pinene | 136.23 | 46.25 | 1.82 | 0.05 | 4.29 | 0.00 | 0.00 | 0.00 | Low |
| ZS52 | deacetyl nomilinic acid | 490.54 | 15.54 | -0.69 | 0.79 | 0.67 | 9.00 | 3.00 | 146.80 | Low |
| ZS53 | ichangin | 488.53 | 16.48 | -0.59 | 0.79 | 0.67 | 9.00 | 2.00 | 135.80 | High |
| ZS54 | citrusin II | 712.79 | 3.08 | -0.72 | 0.25 | -0.98 | 7.00 | 6.00 | 201.91 | Low |
| ZS55 | Kaempferitrin | 578.52 | 8.16 | -2.20 | 0.79 | -2.69 | 14.00 | 8.00 | 228.97 | Low |
| ZS56 | Eriodictin | 434.39 | 2.55 | -0.97 | 0.74 | -1.15 | 10.00 | 6.00 | 166.14 | Low |
| ZS57 | isosakuranetin-7-rutinoside | 608.17 | 11.00 | -2.13 | 0.69 | -0.43 | 9.00 | 4.00 | 134.91 | High |
| ZS58 | loniccrin | 594.52 | 3.84 | -2.34 | 0.73 | -3.43 | 15.00 | 9.00 | 249.20 | Low |
| ZS59 | Neodiosmin | 608.54 | 11.00 | -2.13 | 0.69 | -3.23 | 15.00 | 8.00 | 238.20 | Low |
| ZS60 | Neoeriocitrin | 596.53 | 3.93 | -2.38 | 0.73 | -3.24 | 15.00 | 9.00 | 245.29 | Low |
| ZS61 | neohesperidin | 610.56 | 11.57 | -2.21 | 0.70 | -3.04 | 15.00 | 8.00 | 234.29 | Low |
| ZS62 | poncirin | 594.56 | 36.55 | -1.98 | 0.74 | -2.57 | 14.00 | 7.00 | 214.06 | Low |
| ZS63 | Rutin | 610.52 | 3.20 | -2.60 | 0.68 | -3.89 | 16.00 | 10.00 | 269.43 | Low |
| ZS64 | Diosmin | 608.54 | 12.70 | -2.02 | 0.66 | -3.23 | 15.00 | 8.00 | 238.20 | Low |
| ZS65 | Isorhoifolin | 578.52 | 7.86 | -2.06 | 0.75 | -2.96 | 14.00 | 8.00 | 228.97 | Low |
| ZS66 | Linarin | 592.55 | 39.84 | -1.83 | 0.71 | -2.76 | 14.00 | 7.00 | 217.97 | Low |
| ZS67 | Neoponcirin | 594.56 | 41.24 | -1.90 | 0.71 | -2.57 | 14.00 | 7.00 | 214.06 | Low |
| ZS68 | Lophenol | 400.68 | 8.64 | 1.37 | 0.72 | 6.54 | 1.00 | 1.00 | 20.23 | Low |
| ZS69 | Isosinomenine | 329.39 | 16.90 | 0.67 | 0.46 | 1.30 | 5.00 | 1.00 | 59.00 | High |
| ZS70 | N-methyltyramine | 151.21 | 21.08 | 1.12 | 0.03 | 1.53 | 2.00 | 2.00 | 32.26 | High |
| ZS71 | Monohydryoxy-tetramethoxyflavone | 358.34 | 45.38 | 1.19 | 0.37 | 0.40 | 7.00 | 1.00 | 87.36 | High |
| ZS72 | Tetrahydryoxy-dimethoxy flavone | 346.29 | 15.68 | 0.35 | 0.36 | -0.59 | 8.00 | 4.00 | 129.59 | High |
| ZS73 | Diosmetin | 300.26 | 42.87 | 0.46 | 0.27 | 0.22 | 6.00 | 3.00 | 100.13 | High |
| ZS74 | Diosmetin-7-O-glucoside | 462.40 | 8.01 | -1.14 | 0.82 | -1.89 | 11.00 | 6.00 | 179.28 | Low |
| ZS75 | 5-demethylnobiletin | 388.37 | 89.03 | 1.01 | 0.48 | 0.11 | 8.00 | 1.00 | 96.59 | High |
| ZS76 | Scoparone | 206.19 | 69.62 | 1.01 | 0.09 | 1.05 | 4.00 | 0.00 | 48.67 | High |
| ZS77 | citrusin A | 538.54 | 25.04 | -1.78 | 0.73 | -1.88 | 12.00 | 7.00 | 187.76 | Low |
| ZS78 | Isorhamnetin-3-O-glucoside | 478.40 | 1.17 | -1.34 | 0.80 | -2.37 | 12.00 | 7.00 | 199.51 | Low |
| ZS79 | Chrysoeriol | 300.26 | 41.60 | 0.45 | 0.27 | 0.22 | 6.00 | 3.00 | 100.13 | High |
| ZS80 | Homoeriodictyol | 302.28 | 2.21 | 0.40 | 0.27 | 0.41 | 6.00 | 3.00 | 96.22 | High |
| ZS81 | Xanthotoxin | 216.19 | 36.14 | 1.04 | 0.13 | 1.18 | 4.00 | 0.00 | 52.58 | High |
| ZS82 | Sakuranetin | 286.28 | 40.19 | 0.59 | 0.24 | 0.96 | 5.00 | 2.00 | 75.99 | High |
| ZS83 | Limettin | 206.19 | 28.97 | 0.98 | 0.09 | 1.05 | 4.00 | 0.00 | 48.67 | High |
| ZS84 | citrusin B | 568.57 | 7.84 | -1.56 | 0.71 | -2.16 | 13.00 | 7.00 | 196.99 | Low |
| ZS85 | Acacetin | 284.26 | 37.69 | 0.65 | 0.24 | 0.77 | 5.00 | 2.00 | 79.90 | High |
| ZS86 | Isosakuranetin | 286.28 | 37.59 | 0.58 | 0.24 | 0.96 | 5.00 | 2.00 | 75.99 | High |
| ZS87 | 2,4,6-Trimethoxyphenol-1-O-beta-D-apiofuranosyl-(1-6)-beta-D-glucopyranoside | 478.44 | 20.56 | -1.18 | 0.66 | -3.16 | 13.00 | 6.00 | 185.99 | Low |
| ZS88 | N-Methyl tyramine-O-alpha-L-rhamnopyranoside | 297.35 | 36.70 | -0.04 | 0.19 | -0.16 | 6.00 | 4.00 | 91.18 | High |
| ZS89 | 2,3,4,7,-Tetramethoxyxanthone-1-O-beta-D-xylopyranosyl-(1-6)-beta-D-glucopyranoside | 626.56 | 67.75 | -1.29 | 0.62 | -3.39 | 16.00 | 6.00 | 225.43 | Low |
| ZS90 | citrusin III | 727.85 | 19.12 | -1.20 | 0.30 | -1.54 | 9.00 | 7.00 | 226.58 | Low |
| ZS91 | citrusin IV | 897.93 | 9.28 | -2.35 | 0.16 | -3.27 | 14.00 | 11.00 | 351.70 | Low |
| ZS92 | Acetyl-O-naringin | 622.57 | 10.18 | -1.91 | 0.68 | -2.37 | 15.00 | 7.00 | 231.13 | Low |
| ZS93 | dehydrodiconiferylalcohol-4-β-D-glucoside | 520.53 | 22.61 | -1.41 | 0.87 | -0.72 | 11.00 | 6.00 | 167.53 | Low |
| ZS94 | 2"-O-β-xylosylvitexin | 564.49 | 2.17 | -2.24 | 0.83 | -3.56 | 14.00 | 9.00 | 239.97 | Low |
| ZS95 | Naringenin-4'-glucose-7-neohesperidoside | 742.68 | 3.78 | -3.08 | 0.29 | -4.85 | 19.00 | 11.00 | 304.21 | Low |
| ZS96 | 9-hydroxylinalool-9β-glucopyranoside | 332.39 | 6.01 | -1.01 | 0.22 | -0.48 | 7.00 | 5.00 | 119.61 | High |
| ZS97 | carveol-6β-glucopyranoside | 314.37 | 14.78 | -0.29 | 0.20 | -0.09 | 6.00 | 4.00 | 99.38 | High |
| ZS98 | nobiletin-3-O-β-glucoside | 580.53 | 22.70 | -0.70 | 0.80 | -2.29 | 14.00 | 4.00 | 184.97 | Low |
| ZS99 | (1S,5S)-1-isopropyl-4-methylenebicyclo[3.1.0]hexane | 136.23 | 42.45 | 1.83 | 0.04 | 4.29 | 0.00 | 0.00 | 0.00 | Low |
| ZS100 | (2S)-7-[(2S,3R,4S,5S,6R)-4,5-dihydroxy-6-methylol-3-[(2S,3R,4R,5R,6S)-3,4,5-trihydroxy-6-methyl-tetrahydropyran-2-yl]oxy-tetrahydropyran-2-yl]oxy-5-hydro | 610.56 | 11.17 | -2.27 | 0.70 | -3.04 | 15.00 | 8.00 | 234.29 | Low |
| ZS101 | (R)-(-)-alpha-Phellandrene | 136.23 | 27.51 | 1.86 | 0.02 | 3.27 | 0.00 | 0.00 | 0.00 | Low |
| ZS102 | (Z)-caryophyllene | 204.35 | 30.29 | 1.82 | 0.09 | 4.63 | 0.00 | 0.00 | 0.00 | Low |
| ZS103 | 4',5,7,8-Tetramethoxyflavone | 342.34 | 23.45 | 1.03 | 0.36 | 0.94 | 6.00 | 0.00 | 67.13 | High |
| ZS104 | synephrine | 167.21 | 75.25 | 0.63 | 0.04 | 0.65 | 3.00 | 3.00 | 52.49 | High |
| ZS105 | 4-[(2S,3R)-5-[(E)-3-hydroxyprop-1-enyl]-7-methoxy-3-methylol-2,3-dihydrobenzofuran-2-yl]-2-methoxy-phenol | 358.39 | 50.76 | 0.03 | 0.39 | 1.09 | 6.00 | 3.00 | 88.38 | High |
| ZS106 | 4-[(Z)-3-hydroxyprop-1-enyl]-2,6-dimethoxyphenol | 210.23 | 49.15 | 0.56 | 0.06 | 0.84 | 4.00 | 2.00 | 58.92 | High |
| ZS107 | 5,7,4'-Trimethylapigenin | 312.32 | 39.83 | 1.01 | 0.30 | 1.25 | 5.00 | 0.00 | 57.90 | High |
| ZS108 | Hesperetin | 302.28 | 47.74 | 0.28 | 0.27 | 0.41 | 6.00 | 3.00 | 96.22 | High |
| ZS109 | 6-Methoxy aurapten | 328.40 | 31.24 | 1.01 | 0.30 | 3.16 | 4.00 | 0.00 | 48.67 | High |
| ZS110 | Ammidin | 270.28 | 34.55 | 1.13 | 0.22 | 2.14 | 4.00 | 0.00 | 52.58 | High |
| ZS111 | apigenin | 270.24 | 23.06 | 0.43 | 0.21 | 0.52 | 5.00 | 3.00 | 90.90 | High |
| ZS112 | Auraptene | 298.38 | 25.62 | 1.24 | 0.24 | 3.51 | 3.00 | 0.00 | 39.44 | High |
| ZS113 | citrusin C | 326.34 | 17.59 | -0.49 | 0.23 | -0.54 | 7.00 | 4.00 | 108.61 | High |
| ZS114 | caryophellene | 204.35 | 23.79 | 1.84 | 0.09 | 4.63 | 0.00 | 0.00 | 0.00 | Low |
| ZS115 | naringenin | 272.26 | 59.29 | 0.28 | 0.21 | 0.71 | 5.00 | 3.00 | 86.99 | High |
| ZS116 | citrusin A_qt | 376.40 | 13.00 | -0.42 | 0.36 | 0.68 | 7.00 | 4.00 | 108.61 | High |
| ZS117 | Tetramethoxyluteolin | 342.34 | 43.68 | 0.96 | 0.37 | 0.94 | 6.00 | 0.00 | 67.13 | High |
| ZS118 | citrusin B_qt | 406.43 | 11.65 | -0.35 | 0.42 | 0.38 | 8.00 | 4.00 | 117.84 | High |
| ZS119 | Coniferin | 342.34 | 10.28 | -1.00 | 0.27 | -1.32 | 8.00 | 5.00 | 128.84 | High |
| ZS120 | Coniferol | 180.20 | 44.88 | 0.55 | 0.05 | 1.13 | 3.00 | 2.00 | 49.69 | High |
| ZS121 | Cymol | 134.22 | 27.20 | 1.86 | 0.02 | 4.47 | 0.00 | 0.00 | 0.00 | Low |
| ZS122 | D-Camphene | 136.23 | 34.98 | 1.81 | 0.04 | 4.29 | 0.00 | 0.00 | 0.00 | Low |
| ZS123 | Prangenin | 286.28 | 43.60 | 0.80 | 0.29 | 1.39 | 5.00 | 0.00 | 65.11 | High |
| ZS124 | Rhoifolin | 578.52 | 6.68 | -1.87 | 0.77 | -2.96 | 14.00 | 8.00 | 228.97 | Low |
| ZS125 | didymin | 594.57 | 38.55 | -2.00 | 0.24 | -2.57 | 14.00 | 7.00 |  |  |
| ZS126 | Linoleic Acid | 280.45 | 41.24 | -1.82 | 0.71 | 4.47 | 2.00 | 1.00 | 37.30 | High |
| ZS127 | Eriocitrin | 596.53 | 4.52 | -2.23 | 0.70 | -3.24 | 15.00 | 9.00 | 245.29 | Low |
| ZS128 | Eriodyctiol (flavanone) | 288.25 | 41.35 | 0.05 | 0.24 | 0.16 | 6.00 | 4.00 | 107.22 | High |
| ZS129 | eugenol | 164.20 | 56.24 | 1.35 | 0.04 | 2.01 | 2.00 | 1.00 | 29.46 | High |
| ZS130 | hesperidin | 610.56 | 13.33 | -2.03 | 0.67 | -3.04 | 15.00 | 8.00 | 234.29 | Low |
| ZS131 | isolimonic acid | 639.01 | 48.86 | 0.43 | 0.18 | 4.33 | 3.00 | 1.00 | 57.61 | High |
| ZS132 | isopimpinellin | 246.22 | 25.93 | 0.94 | 0.17 | 0.89 | 5.00 | 0.00 | 61.81 | High |
| ZS133 | Scolymoside | 594.52 | 3.84 | -2.16 | 0.73 | -3.43 | 15.00 | 9.00 | 249.20 | Low |
| ZS134 | Isosinensetin | 372.37 | 51.15 | 1.16 | 0.44 | 0.63 | 7.00 | 0.00 | 76.36 | High |
| ZS135 | Sinensetin | 372.37 | 50.56 | 1.12 | 0.45 | 0.63 | 7.00 | 0.00 | 76.36 | High |
| ZS136 | L-Limonen | 136.23 | 38.09 | 1.83 | 0.02 | 3.27 | 0.00 | 0.00 | 0.00 | Low |
| ZS137 | luteolin | 286.24 | 36.16 | 0.19 | 0.25 | -0.03 | 6.00 | 4.00 | 111.13 | High |
| ZS138 | Majudin | 216.19 | 42.21 | 0.94 | 0.13 | 1.18 | 4.00 | 0.00 | 52.58 | High |
| ZS139 | Moslene | 136.23 | 33.02 | 1.88 | 0.02 | 3.27 | 0.00 | 0.00 | 0.00 | Low |
| ZS140 | Myrcene | 136.23 | 24.96 | 1.84 | 0.02 | 3.56 | 0.00 | 0.00 | 0.00 | Low |
| ZS141 | naringenin-4'-glucoside-7-rutinoside | 742.68 | 3.48 | -3.50 | 0.28 | -4.85 | 19.00 | 11.00 | 304.21 | Low |
| ZS142 | naringenin-4'-glucoside-7-rutinoside_qt | 262.26 | 30.61 | 0.33 | 0.16 | 0.26 | 5.00 | 3.00 | 86.99 | High |
| ZS143 | naringin | 580.53 | 6.92 | -1.99 | 0.78 | -2.77 | 14.00 | 8.00 | 225.06 | Low |
| ZS144 | Narirutin | 580.53 | 8.15 | -1.80 | 0.75 | -2.77 | 14.00 | 8.00 | 225.06 | Low |
| ZS145 | neohesperidin_qt | 302.28 | 71.17 | 0.26 | 0.27 | 0.41 | 6.00 | 3.00 | 96.22 | High |
| ZS146 | nobiletin | 402.39 | 61.67 | 1.05 | 0.52 | 0.34 | 8.00 | 0.00 | 85.59 | High |
| ZS147 | Syrigin | 372.37 | 14.64 | -1.01 | 0.32 | -1.59 | 9.00 | 5.00 | 138.07 | Low |
| ZS148 | tangeretin | 372.37 | 21.38 | 1.23 | 0.43 | 0.63 | 7.00 | 0.00 | 76.36 | High |
| ZS149 | prangenin hydrate | 304.29 | 72.63 | 0.14 | 0.29 | 0.57 | 6.00 | 2.00 | 93.04 | High |
| ZS150 | neohesperidin | 610.62 | 11.57 | -2.05 | 0.69 | -3.04 | 15.00 | 8.00 | 234.29 | Low |
