# Supplementary material for: System Pharmacology-Based Strategy to Decode the Synergistic Mechanism of Zhi-zhu Wan for Functional Dyspepsia
Source: Front Pharmacol. 2018 Aug 6;9:841. doi: 10.3389/fphar.2018.00841 (PMC6087764; doi:10.3389/fphar.2018.00841)
Supplement: Supplementary file 2 [file Table_2.DOCX]

**Table S2** The relationship probability between targets and diseases.

| Disease Id | Disease Name | P value | Mesh |
| --- | --- | --- | --- |
| C0525045 | Mood Disorders | -4.20E-12 | Mental Disorders |
| C0035126 | Reperfusion Injury | -1.21E-12 | Cardiovascular Diseases |
| C0001973 | Alcoholic Intoxication, Chronic | 5.38E-12 | Mental Disorders |
| C0004153 | Atherosclerosis | 1.32E-11 | Cardiovascular Diseases |
| C0011570 | Mental Depression | 4.09E-11 | Behavior and Behavior Mechanisms |
| C1269683 | Major Depressive Disorder | 5.54E-11 | Mental Disorders |
| C0036341 | Schizophrenia | 1.02E-10 | Mental Disorders |
| C0021368 | Inflammation | 2.47E-10 | Pathological Conditions |
| C0038587 | Substance Withdrawal Syndrome | 2.51E-10 | Pathological Conditions |
| C0020538 | Hypertensive disease | 2.93E-10 | Cardiovascular Diseases |
| C0033578 | Prostatic Neoplasms | 4.41E-10 | Neoplasms |
| C1458155 | Mammary Neoplasms | 4.64E-10 | Neoplasms |
| C0007786 | Brain Ischemia | 9.32E-10 | Cardiovascular Diseases |
| C0011581 | Depressive disorder | 1.02E-09 | Mental Disorders |
| C0001418 | Adenocarcinoma | 1.49E-09 | Neoplasms |
| C0024809 | Marijuana Abuse | 4.11E-09 | Mental Disorders |
| C0041696 | Unipolar Depression | 7.29E-09 | Mental Disorders |
| C0011853 | Diabetes Mellitus, Experimental | 1.50E-08 | Endocrine System Diseases |
| C0085762 | Alcohol abuse | 1.66E-08 | Mental Disorders |
| C0236736 | Cocaine-Related Disorders | 1.77E-08 | Mental Disorders |
