# Supplementary material for: System Pharmacology-Based Strategy to Decode the Synergistic Mechanism of Zhi-zhu Wan for Functional Dyspepsia
Source: Front Pharmacol. 2018 Aug 6;9:841. doi: 10.3389/fphar.2018.00841 (PMC6087764; doi:10.3389/fphar.2018.00841)
Supplement: Supplementary file 3 [file Table_3.DOCX]

**Table S3** The value of active ingredients in Zhishi-Baizhu.

| **Component** | **ω_ei_** | **A+B/A-B** | **A_ij_** | **C_i_** | **P_j_** | **CS** |
| --- | --- | --- | --- | --- | --- | --- |
| ZS88 | 0.006163 | 1 | 1.006163 | 4 | 65 | 261.60 |
| ZS86 | 0.023112 | 1 | 1.023112 | 15 | 210 | 3222.80 |
| ZS85 | 0.058552 | 1 | 1.058552 | 38 | 366 | 14722.34 |
| ZS82 | 0.020031 | 1 | 1.020031 | 13 | 183 | 2426.65 |
| ZS79 | 0.046225 | 1 | 1.046225 | 30 | 299 | 9384.64 |
| ZS75 | 0.023112 | 1 | 1.023112 | 15 | 182 | 2793.10 |
| ZS73 | 0.041602 | 1 | 1.041602 | 27 | 304 | 8549.47 |
| ZS71 | 0.024653 | 1 | 1.024653 | 16 | 213 | 3492.02 |
| ZS42 | 0.004622 | 1 | 1.004622 | 3 | 64 | 192.89 |
| ZS41 | 0.010786 | 1 | 1.010786 | 7 | 157 | 1110.85 |
| ZS40 | 0.007704 | 1 | 1.007704 | 5 | 101 | 508.89 |
| ZS39 | 0.006163 | 1 | 1.006163 | 4 | 39 | 156.96 |
| ZS36 | 0.003082 | 1 | 1.003082 | 2 | 39 | 78.24 |
| ZS35 | 0.009245 | 1 | 1.009245 | 6 | 112 | 678.21 |
| ZS34 | 0.004622 | 1 | 1.004622 | 3 | 57 | 171.79 |
| ZS30 | 0.013867 | 1 | 1.013867 | 9 | 112 | 1021.98 |
| ZS28 | 0.009245 | 1 | 1.009245 | 6 | 130 | 787.21 |
| ZS27 | 0.010786 | 1 | 1.010786 | 7 | 104 | 735.85 |
| ZS26 | 0.009245 | 1 | 1.009245 | 6 | 79 | 478.38 |
| ZS25 | 0.006163 | 1 | 1.006163 | 4 | 88 | 354.17 |
| ZS24 | 0.007704 | 1 | 1.007704 | 5 | 96 | 483.70 |
| ZS23 | 0.010786 | 1 | 1.010786 | 7 | 104 | 735.85 |
| ZS22 | 0.015408 | 1 | 1.015408 | 10 | 157 | 1594.19 |
| ZS21 | 0.006163 | 1 | 1.006163 | 4 | 71 | 285.75 |
| ZS150 | 0.01849 | 1 | 1.01849 | 12 | 142 | 1735.51 |
| ZS149 | 0.010786 | 1 | 1.010786 | 7 | 146 | 1033.02 |
| ZS145 | 0.023112 | 1 | 1.023112 | 15 | 202 | 3100.03 |
| ZS144 | 0.013867 | 1 | 1.013867 | 9 | 118 | 1076.73 |
| ZS143 | 0.030817 | 1 | 1.030817 | 20 | 162 | 3339.85 |
| ZS137 | 0.05547 | 1 | 1.05547 | 36 | 346 | 13146.93 |
| ZS135 | 0.030817 | 1 | 1.030817 | 20 | 265 | 5463.33 |
| ZS134 | 0.023112 | 1 | 1.023112 | 15 | 202 | 3100.03 |
| ZS131 | 0.001541 | 1 | 1.001541 | 1 | 2 | 2.00 |
| ZS130 | 0.023112 | 1 | 1.023112 | 15 | 130 | 1995.07 |
| ZS128 | 0.027735 | 1 | 1.027735 | 18 | 235 | 4347.32 |
| ZS123 | 0.010786 | 1 | 1.010786 | 7 | 133 | 941.04 |
| ZS117 | 0.041602 | 1 | 1.041602 | 27 | 290 | 8155.75 |
| ZS115 | 0.030817 | 1 | 1.030817 | 20 | 236 | 4865.45 |
| ZS110 | 0.012327 | 1 | 1.012327 | 8 | 167 | 1352.47 |
| ZS109 | 0.012327 | 1 | 1.012327 | 8 | 98 | 793.66 |
| ZS108 | 0.003082 | 1 | 1.003082 | 2 | 4 | 8.02 |
| ZS107 | 0.043143 | 1 | 1.043143 | 28 | 311 | 9083.69 |
| ZS105 | 0.015408 | 1 | 1.015408 | 10 | 74 | 751.40 |
| ZS104 | 0.021572 | 1 | 1.021572 | 14 | 90 | 1287.18 |
| BZ100 | 0.072072 | 1 | 1.072072 | 8 | 88 | 754.74 |
| BZ102 | 0.045045 | 1 | 1.045045 | 5 | 80 | 418.02 |
| BZ107 | 0.072072 | 1 | 1.072072 | 8 | 20 | 171.53 |
| BZ110 | 0.063063 | 1 | 1.063063 | 7 | 121 | 900.41 |
| BZ119 | 0.081081 | 1 | 1.081081 | 9 | 53 | 515.68 |
| BZ124 | 0.018018 | 1 | 1.018018 | 2 | 10 | 20.36 |
| BZ125 | 0.054054 | 1 | 1.054054 | 6 | 84 | 531.24 |
| BZ27 | 0.063063 | 1 | 1.063063 | 7 | 55 | 409.28 |
| BZ42 | 0.027027 | 1 | 1.027027 | 3 | 26 | 80.11 |
| BZ57 | 0.081081 | 1 | 1.081081 | 9 | 78 | 758.92 |
| BZ59 | 0.09009 | 1 | 1.09009 | 10 | 37 | 403.33 |
| BZ60 | 0.054054 | 1 | 1.054054 | 6 | 58 | 366.81 |
| BZ64 | 0.072072 | 1 | 1.072072 | 8 | 88 | 754.74 |
| BZ72 | 0.063063 | 1 | 1.063063 | 7 | 22 | 163.71 |
| BZ75 | 0.063063 | 1 | 1.063063 | 7 | 114 | 848.32 |
| BZ83 | 0.045045 | 1 | 1.045045 | 5 | 45 | 235.14 |
| BZ84 | 0.036036 | 1 | 1.036036 | 4 | 57 | 236.22 |
