# Supplementary figures and images for: System Pharmacology-Based Strategy to Decode the Synergistic Mechanism of Zhi-zhu Wan for Functional Dyspepsia
Source: Front Pharmacol. 2018 Aug 6;9:841. doi: 10.3389/fphar.2018.00841 (PMC6087764; doi:10.3389/fphar.2018.00841)

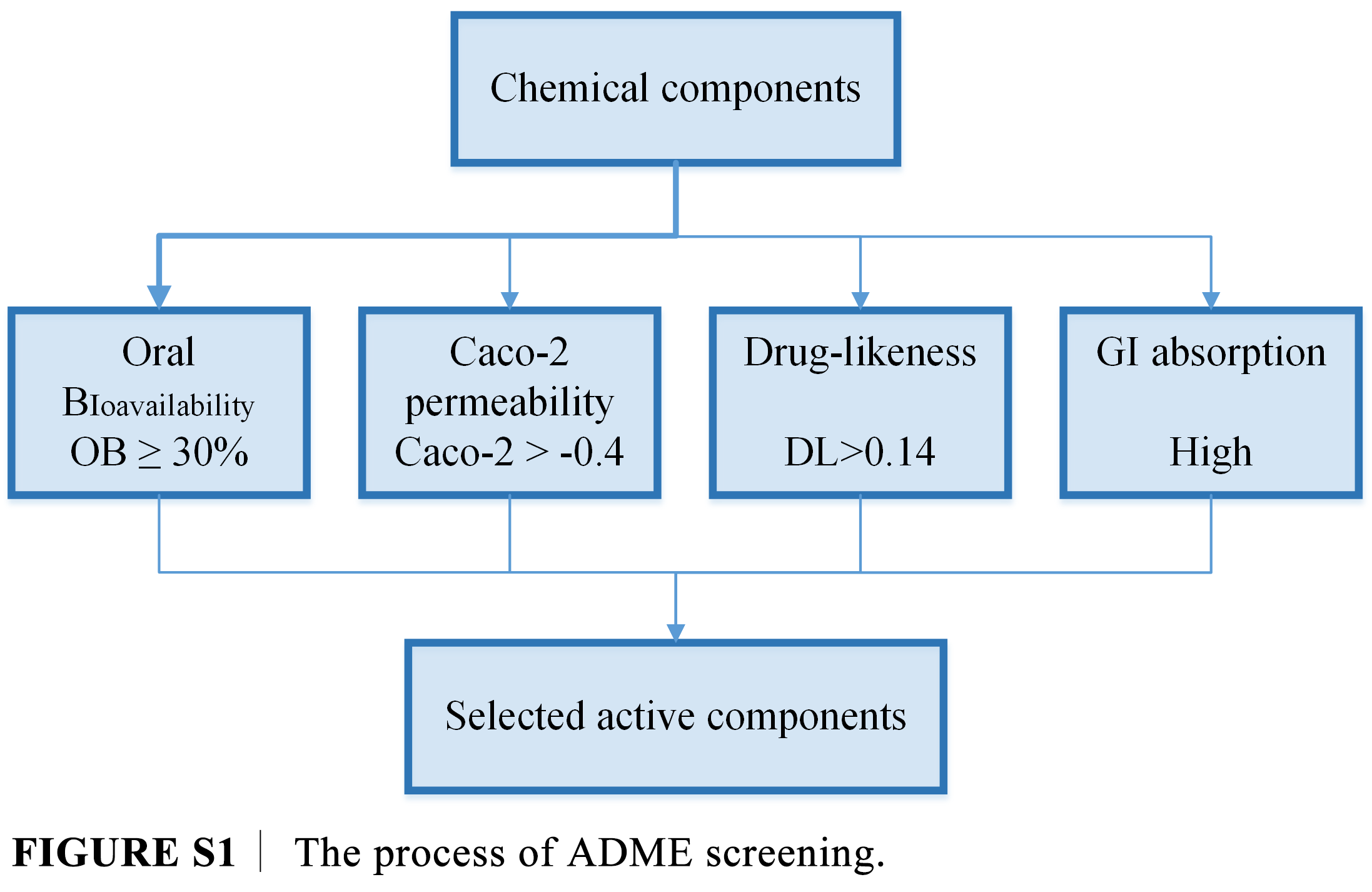

Supplement: Supplementary file 5 [file Image_1.tif]

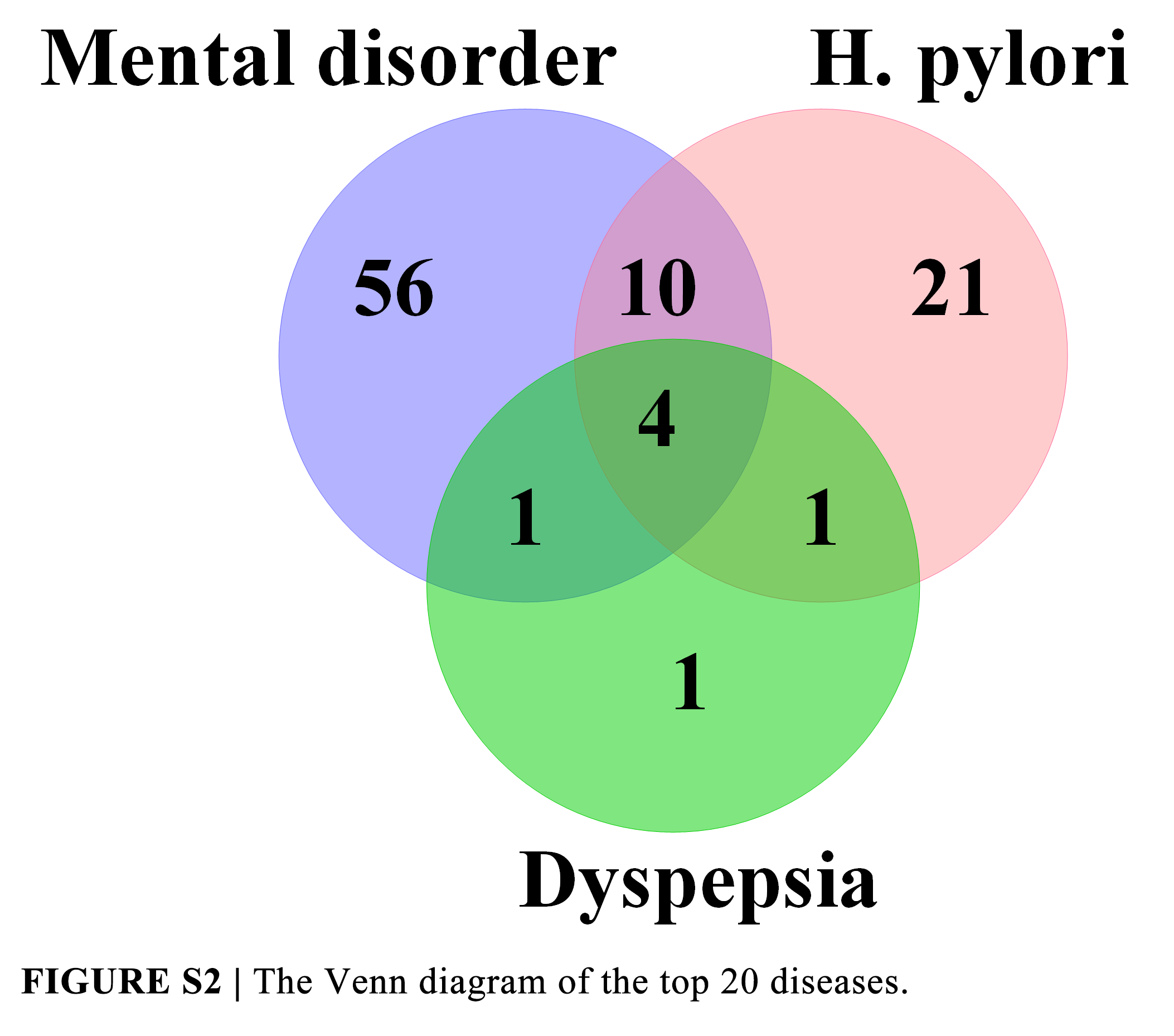

Supplement: Supplementary file 6 [file Image_2.tif]

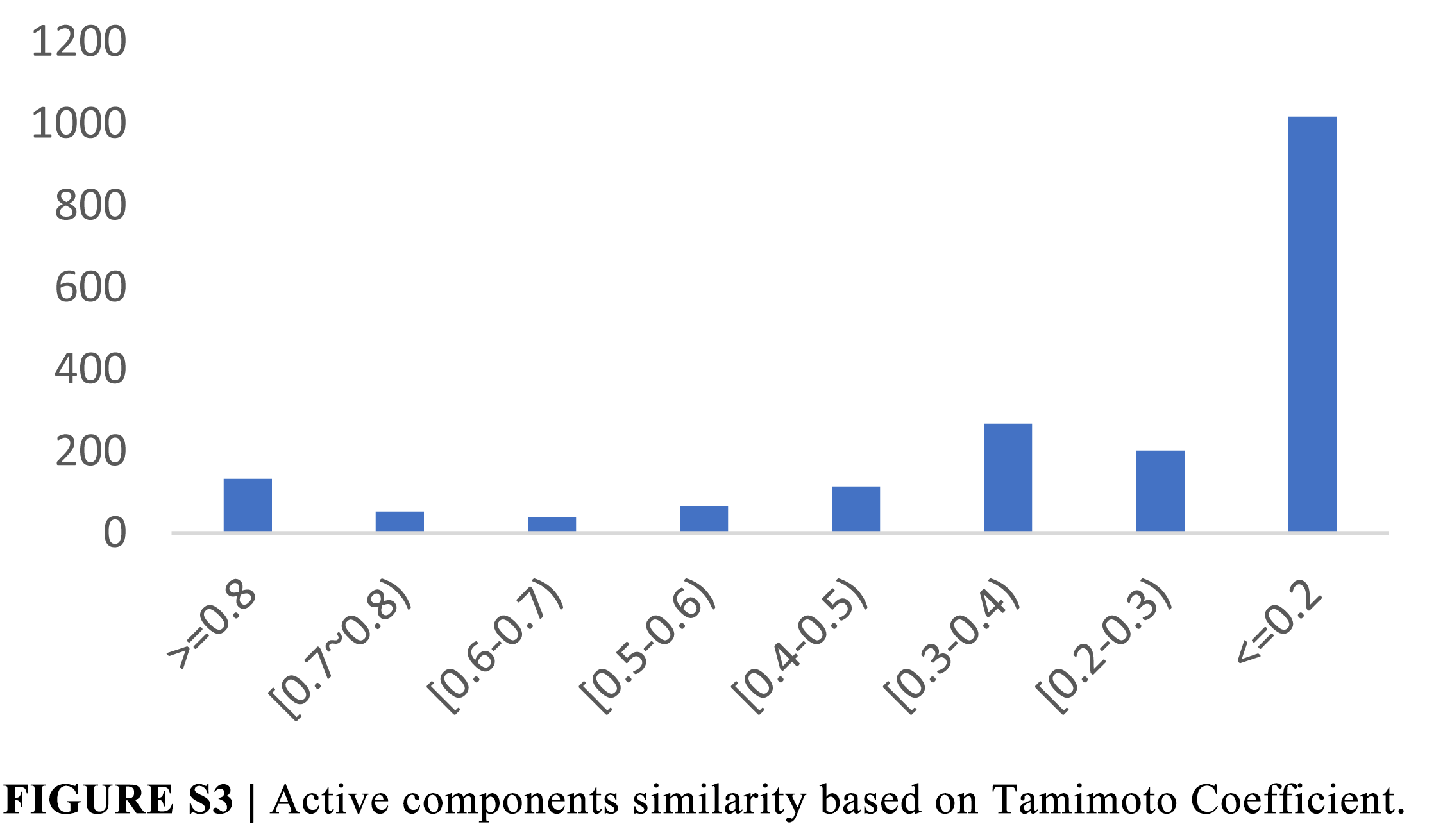

Supplement: Supplementary file 7 [file Image_3.tif]

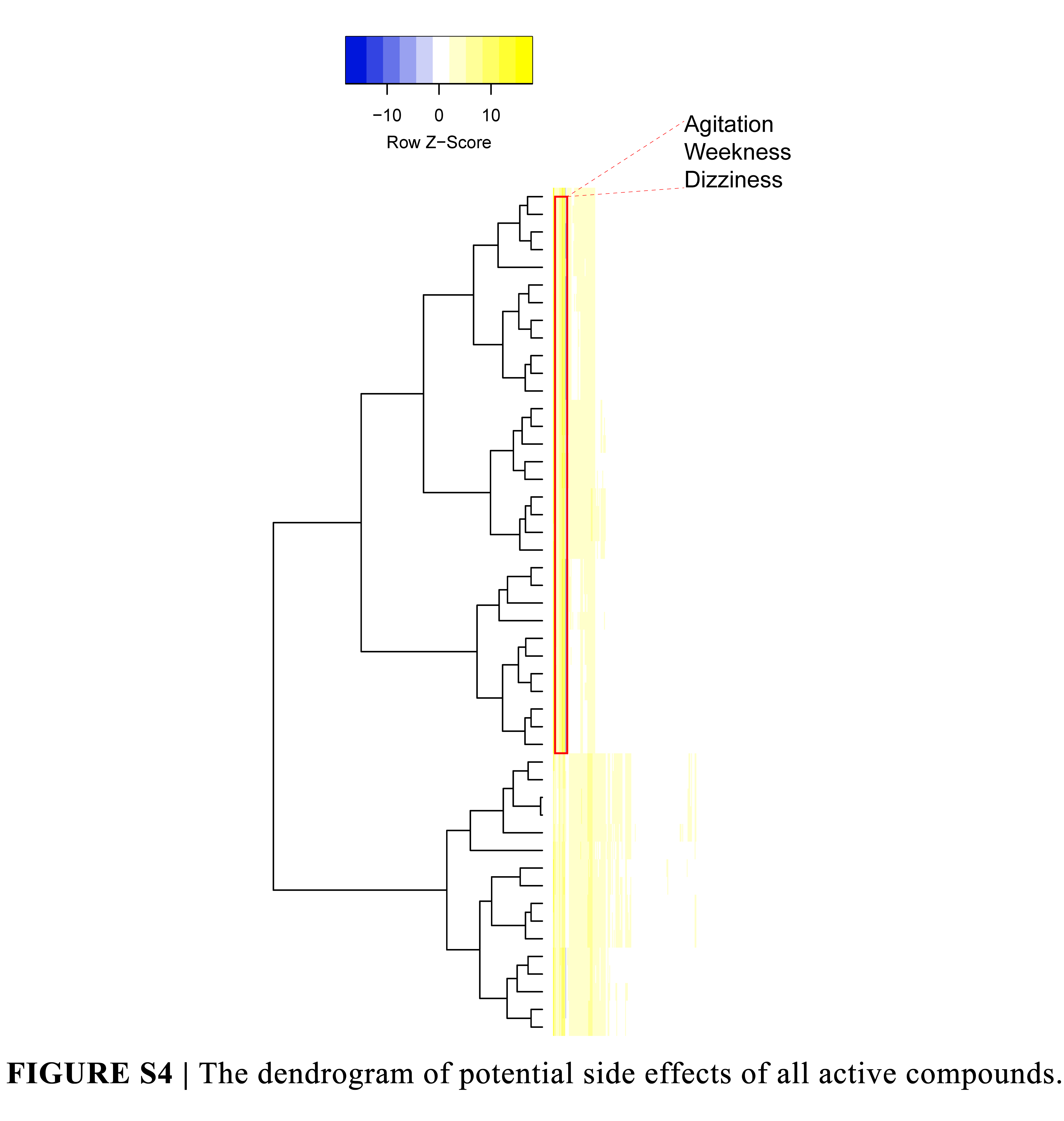

Supplement: Supplementary file 8 [file Image_4.tif]
